# Distinct metabolic patterns of neuropsychiatric systemic lupus erythematosus on hierarchical cluster analysis

**DOI:** 10.1007/s00259-025-07391-z

**Published:** 2025-06-10

**Authors:** Bianca Dagmar Berndorfler, James Mathew Warwick, Patrick Dupont, Riette du Toit, Amori Engelbrecht, Thabiet Jardine, Prabash Sadhai, Tholakele Sabela, Vivian Anopuechi-Clarkson, Alex Govert George Doruyter

**Affiliations:** 1 Division of Nuclear Medicine, Faculty of Medicine and Health Sciences, Stellenbosch University, Cape Town, South Africa; 2 Department of Neurosciences, KU Leuven Brain Institute, Leuven, Belgium; 3 Division of Rheumatology, Department of Medicine, Stellenbosch University, Cape Town, South Africa; 4 NuMeRI Node for Infection Imaging, Central Analytical Facilities, Stellenbosch University, Cape Town, South Africa

**Keywords:** Neuropsychiatric systemic lupus erythematosus (NPSLE), FDG PET brain imaging, Hierarchical cluster analysis, Metabolic brain patterns, Autoimmune encephalitis, Neuroinflammation biomarkers

## Abstract

**Purpose:**

Neuropsychiatric systemic lupus erythematosus (NPSLE) is the second leading cause of morbidity and mortality in systemic lupus erythematosus (SLE). The pathogenesis of NPSLE is poorly understood and diagnosis is difficult. Advances in neuroimaging show promise, but no patterns specific for the disease have emerged. The identification of such patterns may be facilitated by statistical techniques such as cluster analysis.

**Materials and methods:**

FDG PET-CT scans of 46 patients confirmed with NPSLE were included. Median values of each brain region of the Brainnetome atlas were extracted after normalisation for global counts. Hierarchical clustering of patients was performed and demographic, clinical and laboratory characteristics of the clusters were compared.

**Results:**

Our analysis revealed an optimal cluster number of two. Comparing imaging features of scans in patient cluster 1 (*n* = 23) and patient cluster 2 (*n* = 23), scans in the latter group demonstrated significantly higher metabolism in basal ganglia, thalami, medial temporal lobes and cerebellum and hypometabolism in the frontal, parietal, occipital and lateral temporal lobes. Patients in cluster 2 had higher SLEDAI-2 K disease activity scores and erythrocyte sedimentation rates, greater frequencies of psychosis, cerebrovascular accidents, and vasculitis as manifested by rash and mucosal ulcers, and higher rates of positive anti-dsDNA and anti-Smith antibodies, as well as higher anti-Smith antibody titres.

**Conclusion:**

We identified a subgroup of NPSLE patients with a distinct FDG uptake pattern, higher disease activity and higher incidence of certain clinical symptoms and laboratory findings. This metabolic pattern is similar to that described in autoimmune encephalitis and may be driven by a common pathological mechanism.

**Supplementary Information:**

The online version contains supplementary material available at 10.1007/s00259-025-07391-z.

## Introduction

Neuropsychiatric systemic lupus erythematosus (NPSLE) refers to the neurological and psychiatric manifestations of systemic lupus erythematosus (SLE), and affects 15–95% of patients with SLE, depending on the population studied [1]. These manifestations may involve both the central and peripheral nervous systems, contributing to the morbidity and mortality associated with SLE. NPSLE is considered the second leading cause of death in patients with SLE and significantly impacts their quality of life and functional status [2].

The pathogenesis of NPSLE remains poorly understood, complicating both its diagnosis and management [3]. Advances in neuroimaging show promise in assisting with diagnosis, but no patterns specific for the disease have emerged [4]. This is likely due to the heterogeneity of the disease, which may present with diverse clinical symptoms and manifest various laboratory abnormalities [1] depending on underlying pathologies.

In the brain, NPSLE causes focal and non-focal neurological deficits ranging from headaches to seizures and psychosis. Distinguishing NPSLE manifestations from comorbid disorders or medication effects (present in up to 40% of cases) may be difficult [5]. Structural MRI is useful for the evaluation of central focal deficits, detecting ischaemia and infarction thought to be caused by immune complex-induced vasculitis, thrombosis related to phospholipid antibodies [6] and inflammation mediated accelerated atherosclerosis [4]. Whereas focal manifestations of NPSLE are strongly linked to thromboembolic events, the pathogenesis of diffuse, non-focal manifestations remains unclear. Theories focusing on brain-reactive autoantibodies, cytokines, and cell-mediated inflammation have failed to provide a unifying model to explain the pathogenesis of diffuse NPSLE. It has been posited that several pathogenic mechanisms may exist [2]. Symptoms are likely to be caused by microstructural damage and metabolic impairments of neurons, as well as microvascular involvement, which is found in up to 42% of SLE patients with neurological symptoms [2, 4].

Fluorine-18 fluoro-2-deoxyglucose (FDG) PET shows promise in this context, because it can identify alterations in cellular metabolism prior to anatomical changes being detectable on structural imaging [7]. The promise of potentially increased sensitivity with FDG PET suggests a possible role for PET in obtaining earlier and/or more accurate NPSLE diagnoses. While several studies have investigated this, the use of PET-CT generally remains ancillary in NPSLE diagnosis in the clinical setting [8].

Previous studies have predominantly considered NPSLE as a single entity. Given the multiple posited NPSLE mechanisms, it is however plausible that distinct patterns of metabolic abnormalities may exist, which in turn may correlate with specific neuropsychiatric phenotypes. No comprehensive, data-driven analysis has yet been conducted to investigate this.

This study aimed to explore metabolic brain patterns on FDG PET in NPSLE by utilizing a hierarchical clustering algorithm aimed at segregating patients before further characterizing clusters of patients in terms of clinical and laboratory features.

## Materials and methods

### Patient selection and data capturing

This study was approved by the Stellenbosch University Health Research Ethics Committee (reference number N22/11/129). All patients with suspected NPSLE who underwent FDG PET-CT at Tygerberg Hospital between December 2016 and January 2023 were eligible for inclusion. Patients were excluded if there was missing laboratory or clinical data; if they were younger than 16 years old; or when on clinical record review, they did not meet criteria for NPSLE diagnosis. All included patients met diagnostic criteria for NPSLE according to the EULAR/ACR classification criteria for SLE and American College of Rheumatology (ACR) nomenclature and case definitions for central NPSLE [9, 10].

Patient demographics, clinical features of SLE as well as the SLE disease activity index for each patient (SLEDAI-2 K [11]) and details of neurological manifestations according to standard ACR definitions were documented. Routine laboratory investigations as well as serological markers including autoantibodies, complement and inflammatory markers were captured. Chronic medication use in the month preceding the PET-CT was also recorded.

### Image acquisition and processing

Patients fasted for at least 4 h prior to the PET scan and were required to have a blood glucose of less than 10 mmol/l (180 mg/dl) at time of FDG injection [12]. Intravenous lines were placed at least 10 min prior to FDG injection to allow for accommodation. All patients reclined in a quiet dimly lit cubicle, were instructed not to speak, read or be otherwise active 5 min before and 20 min after FDG administration. FDG 150 MBq (± 10%) was injected intravenously in all adult patients, in accordance with the EANM guideline [13]. The dose for three patients between 16 and 17 years of age was adjusted according to the EANM dosage card [14]. The delay between radiopharmaceutical injection and imaging start (uptake time) ranged between 30 and 52 min. No sedation was used for any of the patients. The patients were scanned in a supine position, arms at their side and with their heads stabilized in a head holder. All scans were obtained on a Philips Gemini TF Big-Bore PET/CT scanner. A10-minute dynamic PET acquisition was performed in list mode, as well as a low-dose CT scan for attenuation correction. A dynamic reconstruction of the PET data (rebinned into 5 frames of 2 min each) was performed to assess patient motion. For interpretation purposes and used in this analysis, a static reconstruction (2 mm isotropic voxels, 128 × 128 slice matrix) of the full 10-minute PET acquisition was performed using LOR-RAMLA (15 iterations) with standard corrections for attenuation, scatter, randoms, scatter, dead-time, normalization, and decay.

### Data analysis

PET brain images underwent routine quality control checks as part of the clinical workflow. These include qualitative review of dynamic data to detect excessive motion, visual review of the static reconstructions for motion artefacts (blurring, appearance of double image/ghosting on PET reconstruction), misregistration between the PET and CT acquisitions, and visual check for reconstruction artefacts (attenuation correction, scatter correction). No quantitative measures of image quality were assessed. DICOM files were converted to NIfTI using MRIcroGL v1.2.20220720 [11] and then underwent preprocessing and further analysis in MATLAB 2022b and SPM12: scans were normalized for whole brain counts (to generate relative uptake image) and spatially normalized with the OldNorm option in SPM, using the default values. No smoothing was applied except for standard smoothing during the warping (for spatial normalization). Using the Brainnetome atlas for the cerebrum (comprising 246 subregions across both hemispheres [15]) and the Automated Anatomical Labeling (AAL) atlas for segmentation in the cerebellum (comprising 26 cerebellar subregions [16]), median values in each region were extracted. Hierarchical clustering employing Ward’s linkage method with a Euclidean distance was performed using a custom MATLAB script. To test robustness of our results, additional analyses were performed; namely using the same clustering technique utilizing only the AAL atlas for region definition, and performing a k-means clustering with the original regions (Online Resource 1–4). There was good agreement between results from the additional analyses and our original results, as evident by their DICE coefficients (Online Resource 1–4). A region-based comparison of relative uptake between clusters was performed using one-way ANOVA and two-sample t-tests with statistical threshold of *p* < 0.05 (false discovery rate corrected). Demographic, clinical and laboratory characteristics of the patient clusters were compared using standard statistical methods with statistical threshold of *p* < 0.05: categorical variables were compared using chi-squared tests while Wilcoxon rank-sum tests were performed for comparison of continuous variables. No corrections for multiple comparisons were made in the comparison of clinical and laboratory characteristics between clusters. FDR correction was applied in correlations between the FDG values in each region and the composite disease activity score, SLEDAI-2 K. Additional linear regressions (p threshold < 0.05) were performed to test for relationships between uptake in composite volumes (VOIs comprising respectively all regions where cluster 2 > cluster 1 and all regions where cluster 2 < cluster 1) and SLEDAI-2 K scores, regardless of cluster membership.

## Results

Fifty-six (*n* = 56) FDG PET-CT scans were performed for the stated indication of NPSLE during the period under review. Of these, one patient (*n* = 1) was excluded based on missing data, three (*n* = 3) were excluded on the basis of age < 16 years; four (*n* = 4) were excluded due to non-fulfilment of EULAR/ACR classification criteria for SLE diagnosis [9]; and two (*n* = 2) were excluded due to absence of NPSLE features on clinical record review. The remaining patients (*n* = 46 of which 44 female) were included, with a median age of 31.1 years (IQR: 23.7–43.6 years) (Table 1).

**Table 1:** Demographic, clinical and laboratory characteristics of cluster 1 and 2. Statistically significant p-values indicated in bold. Median (interquartile range) indicated for continuous variables, otherwise patient numbers given

	CLUSTER 1	CLUSTER 2	*p*-value (uncorrected)
*n* (%)	23 (50%)	23 (50%)	
Age at scan (years)	33 (26–41)	28 (23–44)	0.586
Sex, female (%)	23 (100%)	21 (91%)	0.148
Duration of disease (months)	11 (3–81)	4 (1–44)	0.170
SLEDAI-2 K score	**18 (10–26)**	**27 (21–32)**	**0.004**
Symptoms			
Seizures	7	10	0.359
Psychosis	**2**	**8**	**0.02**
Organic brain syndrome^a^	13	8	0.139
Lupus headache	4	1	0.155
Stroke	**0**	**4**	**0.036**
Peripheral neuropathy	0	1	0.312
Transverse myelitis	0	1	0.312
Vasculitis (skin/nail/systemic)	**1**	**6**	**0.04**
Arthritis	5	5	1
Avascular necrosis	0	0	n/a
Myositis	2	2	1
Rash	**6**	**15**	**0.008**
Alopecia	6	11	0.127
Mucosal ulcers	**0**	**5**	**0.018**
Pleurisy/pleuritis	1	3	0.295
Pneumonitis	1	1	1
Interstitial lung disease	0	0	n/a
Pulmonary hypertension	1	2	0.55
Lupus myocarditis	1	5	0.08
Pericarditis/pericardial effusion	2	3	0.636
Libman sacs endocarditis	1	0	0.312
Lupus nephritis	9	11	0.552
Blood results			
White cell count (x 10^9^/L)	4.76 (3.88–5.94)	5.08 (3.39–7.29)	0.878
Absolute lymphocyte count (x 10^9^/L)	1.18 (0.76–1.88)	1.14 (0.76–1.42)	0.98
Haemoglobin (g/dL)	**10.9 (9.75–13.28)**	**9.7 (8.3–11.2)**	**0.032**
Platelets (x 10^9^/L)	245 (180–298)	219 (161–304)	0.538
Serum creatinine (umol/L)	59 (54–77)	69 (59–77)	0.243
eGFR (mL/min/1.73 m^2^)	106 (96–127)	105 (89–114)	0.416
Urine protein: creat ratio (g/mmol creat)	0.13 (0.06–0.22)	0.4 (0.13–0.74)	0.072
C-reactive protein (mg/L)	2 (1–26)	8 (6–53)	0.054
Erythrocyte sedimentation rate (mm/hr)	**27 (15–40)**	**82 (39–130)**	**0.029**
ANA^b^ positivity	18	24	0.842
ANA titre	1:640 (1:320-1:1280)	1:1280 (1:320-1:1280)	0.415
Anti dsDNA^c^ antibody positivity	**12**	**20**	**0.032**
Anti dsDNA titre (IU/ml)	117 (16–201)	187 (46–201)	0.245
aSm^d^ antibody positivity	**5**	**14**	**0.015**
aSm^d^ antibody titre (U/mL)	**12 (2–36)**	**40 (12–145)**	**0.028**
Low complement (C3/C4)	10	18	0.121
ACA^e^ positivity	4	2	0.339
Lupus antibody positivity	4	9	0.172
aß2GP1^f^ antibody positivity	4	2	0.325
Treatment received (during 1 month prior to scan)			
Chloroquine	23	23	1
Prednisone ≤ 10 mg/day	9	8	0.76
Prednisone 10–40 mg/day	6	2	0.12
Prednisone ≥ 40 mg/day	4	9	0.102
IV Solumedrol (pulse 3 days)	3	3	1
Cyclophosphamide oral/IV	2	0	0.148
Mycophenolate Mofetil (MMF)	4	3	0.681
Azathioprine	3	1	0.295

^a^ Altered mental function with impaired orientation, memory, or other intellectual function (with rapid onset and fluctuating clinical features), inability to sustain attention to environment, and ≥ 2 of the following: perceptual disturbance, incoherent speech, insomnia or daytime drowsiness, and increased or decreased psychomotor activity; exclude metabolic, infectious, or drug causes [11].

^b^ Anti-nuclear antibody

^c^ Anti double stranded DNA antibody

^d^ Anti-Smith antibody

^e^ Anti-cardiolipin antibody

^f^ Antibodies against beta-2-glycoprotein-1

**Fig. 1:**
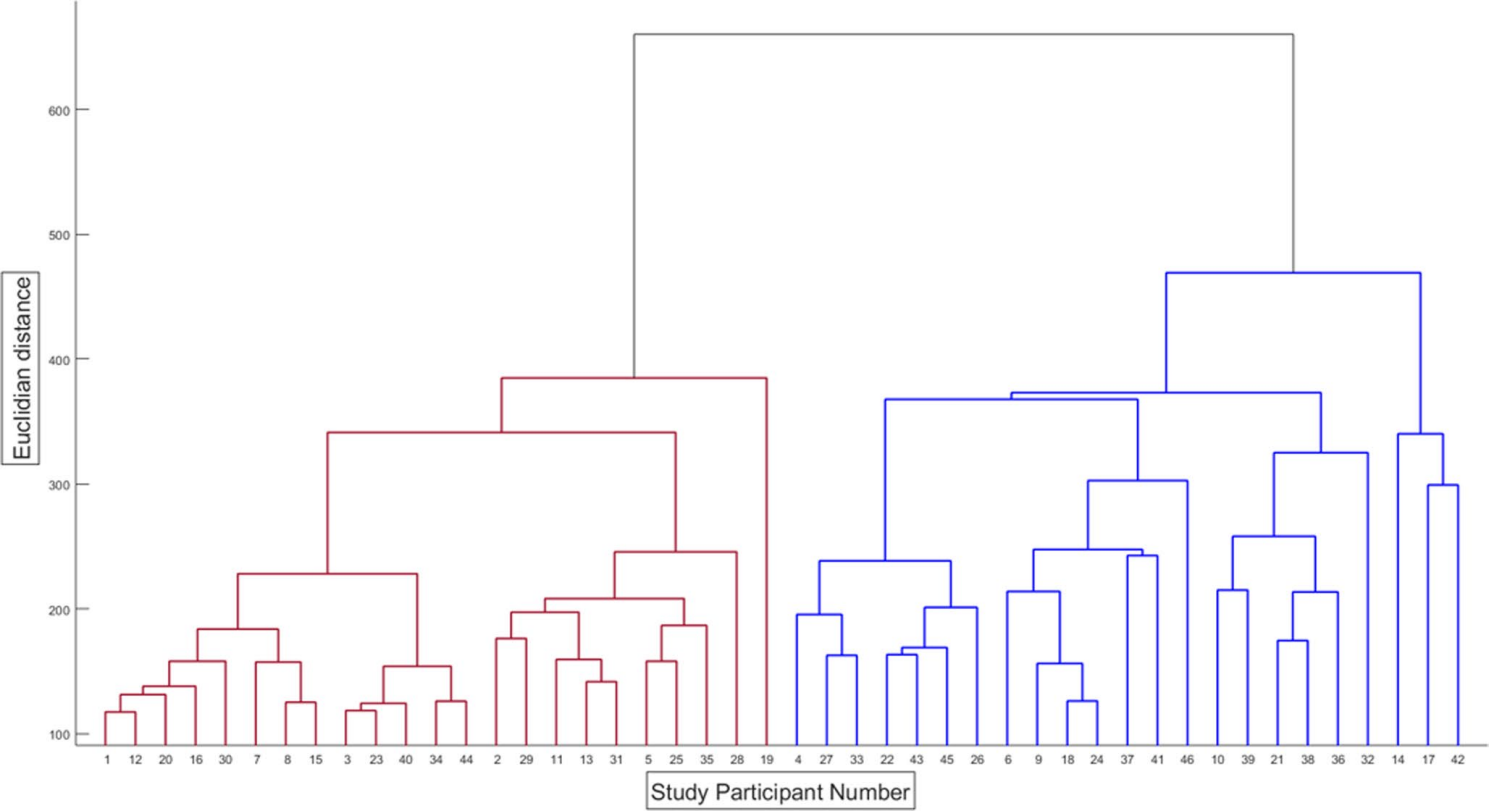
Dendrogram of hierarchical clustering analysis using Ward’s linkage method with a Euclidian distance demonstrating cluster 1 (red) and cluster 2 (blue)

The dendrogram produced by the clustering analysis is shown in Fig. 1. To determine the optimal number of clusters in this hierarchical clustering analysis, the number of clusters was varied between 2 and 10. Five different criteria were evaluated: the gap statistic, the Calinski-Harabasz index, the Silhouette score, the Davies-Bouldin index and the elbow method. For the first 4, the *evalclusters* function in Matlab was utilized to calculate the optimal k. For the elbow method, the within-cluster sum of squared distances between each point and the centroid of its cluster (WCSS) were calculated. The optimal number of clusters was then determined by taking the difference of the difference (= acceleration) to more objectively determine the optimal number of clusters (for more details on this, please refer to Online Resource 5). Based on visual analysis, an optimal cluster number of 2 was selected, which was supported by results of three statistical tests, namely the Elbow method, the Gap Statistic and the Calinski-Harabasz Index (Online Resource 5). Cluster 1 and cluster 2 were each comprised of 23 patients.

**Fig. 2:**
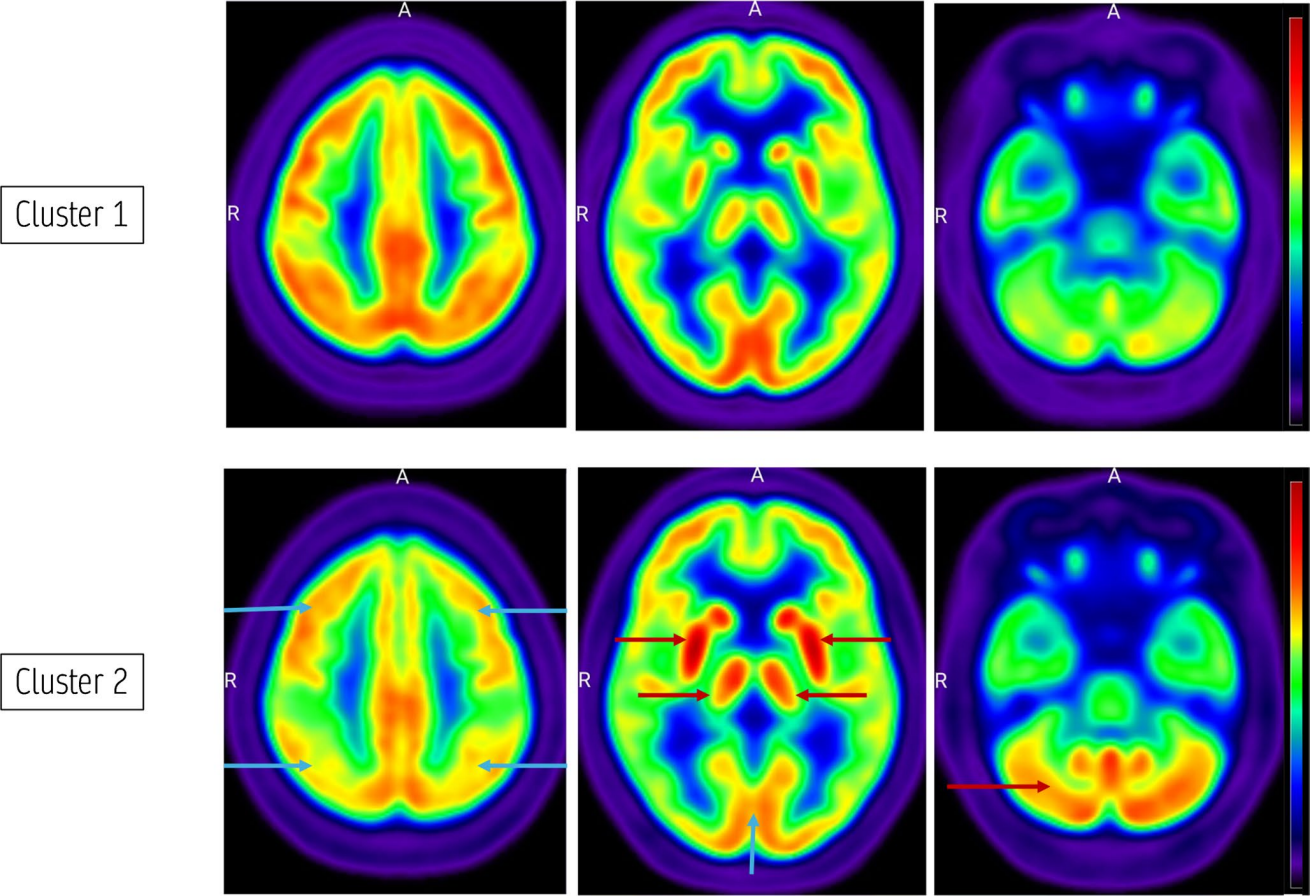
Mean images (voxel-based) of clusters 1 and 2 displayed in NIH colour scale, MRIcroGL [17]. Cluster 2 exhibited visually higher mean metabolism in bilateral striata, thalami and cerebellum (red arrows) and lower mean metabolism in widespread cortical regions (blue arrows) than that in cluster 1. This was confirmed on statistical analysis

Visual inspection of the mean FDG PET image of clusters 1 and 2 revealed several differences (Fig. 2). Compared to cluster 1, cluster 2 had higher mean metabolism in the striata and thalami bilaterally, as well as in the cerebellum. Cluster 2 also demonstrated lower mean metabolism in widespread cortical regions including frontal, parietal and occipital cortices bilaterally compared to cluster 1.

**Fig. 3:**
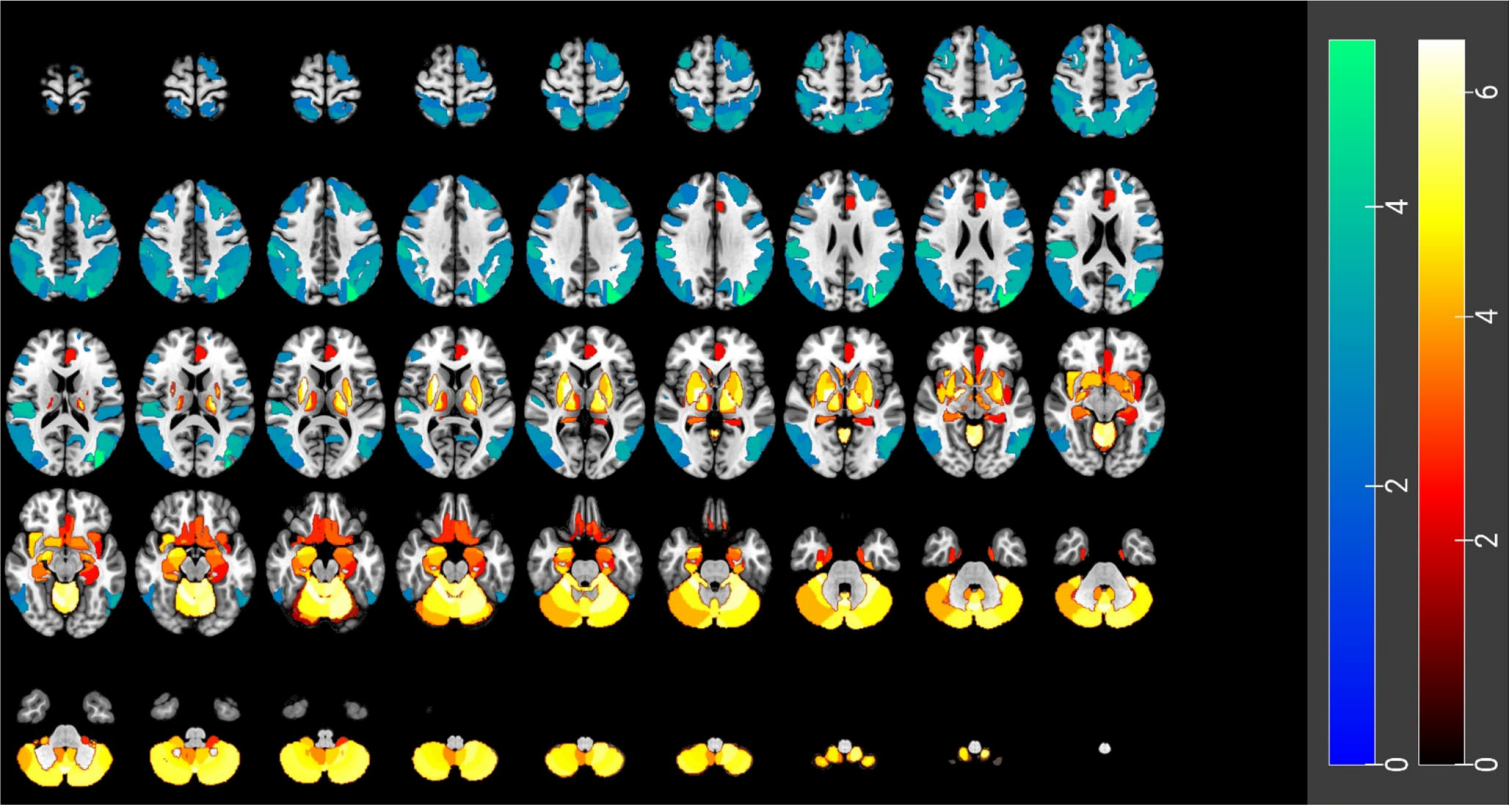
Statistical results: Statistical region-based comparison showing a significant difference in metabolism (FDR corrected *p* < 0.05), highlighting areas where cluster 2 differed significantly from cluster 1. Cluster 2 showed higher metabolism in bilateral basal ganglia, thalami, medial temporal lobes, amygdalae, hippocampi, insular cortices and cerebellum than cluster 1 and lower metabolism in widespread cortical regions including frontal, parietal, occipital and lateral temporal. Increases (red/yellow, “4 hot” colour scale) and decreases (blue/turquoise, “5winter” colour scale), overlaid on MRI template (MNI152) in MRIcroGL [17]. The colour scale represents t-values. Abbreviation: FDR = false discovery rate

The visual findings were confirmed by statistical analysis (Table 2; Fig. 3) which showed significantly higher metabolism in bilateral basal ganglia, bilateral thalami, medial temporal lobes and the cerebellum (FDR corrected *p* < 0.05) in cluster 2 compared to cluster 1. Higher metabolism was also seen in bilateral inferomedial orbital cortices, bilateral amygdalae, bilateral hippocampi, the left anterior cingulate cortex and bilateral insular cortices in cluster 2 compared to cluster 1 (FDR corrected *p* < 0.05). In addition, widespread cortical regions showed lower metabolism in cluster 2 compared to cluster 1, including bilateral frontal, parietal, occipital and lateral temporal cortices.

**Table 2:** Statistical region-based comparison showing a significant difference in metabolism (FDR corrected *p* < 0.05). Region numbers of the cerebrum refer to the brainnetome atlas [14] and for the cerebellum the automated anatomical labeling (AAL) atlas [15]. For atlas descriptors, please refer to supplementary information. (FDR = false discovery rate)

*Contrast*	*Anatomical location*			Hemisphere	Region label ID	*p*-value (uncorrected)
Cluster 2 < cluster 1	Frontal lobe	Superior frontal gyrus	Medial area 8	Left	1	7.12e-03
			Dorsolateral area 8	Left	3	8.71e-04
			Lateral area 9	Left	5	6.71e-03
			Dorsolateral area 6	Left	7	3.80e-03
		Middle frontal gyrus	Dorsal area 9/46	Left	15	1.83e-03
				Right	16	8.74e-03
			Ventrolateral area 8	Left	23	5.05e-04
				Right	24	2.14e-03
			Ventrolateral area 6	Left	25	9.37e-04
				Right	26	3.23e-04
		Inferior frontal gyrus	Dorsal area 44	Left	29	6.64e-03
			Inferior frontal sulcus	Right	32	2.99e-03
		Precentral gyrus	Caudal dorsolateral area 6	Left	55	4.42e-03
			Caudal ventrolateral area 6	Left	63	3.68e-03
				Right	64	5.39e-03
	Temporal lobe	Superior temporal gyrus	Area 41/42	Right	72	3.53e-04
		Middle temporal gyrus	Dorsolateral area 37	Left	85	2.90e-03
				Right	86	3.00e-03
		Inferior temporal gyrus	Extreme lateroventral area 37	Left	91	2.20e-03
				Right	92	6.58e-03
			Ventrolateral area 37	Left	97	4.17e-04
				Right	98	6.90e-03
	Parietal lobe	Superior parietal lobule	Rostrol area 7	Left	125	1.13e-03
				Right	126	1.43e-03
			Caudal area 7	Left	127	5.55e-04
				Right	128	2.60e-04
			Lateral area 5	Left	129	4.34e-03
				Right	130	3.34e-03
			Postcentral area 7	Left	131	8.37e-03
				Right	132	9.78e-03
			Intraparietal area 7	Right	133	2.99e-04
			Caudal area 39	Left	135	3.87e-06
				Right	136	2.50e-03
			Retrodorsal area 39	Left	137	3.79e-04
				Right	138	5.10e-04
			Rostrodorsal area 40	Left	139	1.67e-03
				Right	140	7.03e-04
			Caudal area 40	Left	141	8.24e-04
				Right	142	4.25e-03
			Rostroventral area 39	Left	143	3.65e-04
				Right	144	3.09e-03
			Rostroventral area 40	Left	145	2.34e-03
				Right	146	1.36e-04
		Precuneus	Medial area 7	Left	147	5.30e-04
				Right	148	6.05e-04
			Medial area 5	Left	149	6.31e-03
			Dorsomedial parietooccipital sulcus	Left	151	2.15e-03
		Postcentral gyrus	Area 2	Left	159	2.86e-03
				Right	160	2.49e-03
	Occipital lobe	Lateral occipital cortex	Middle occipital gyrus	Right	200	9.15e-03
			Area V5/MT	Left	201	5.14e-04
				Right	202	3.67e-03
			Lateral superior occipital gyrus	Left	209	5.53e-03
				Right	210	6.08e-03
Cluster 2 > cluster 1	Frontal lobe	Orbital gyrus	Area 13	Left	49	1.02e-03
				Right	50	3.66e-03
	Temporal lobe	Parahippocampal gyrus	Rostrol area 35/36	Right	110	6.60e-03
			Caudal area 35/36	Left	111	8.54e-05
				Right	112	1.27e-05
			Area TL (lateral PPHC, posterior parahippocampal gyrus)	Left	113	8.59e-03
				Right	114	1.62e-04
			Area 28/34 (EC, entorhinal cortex)	Left	115	2.67e-03
				Right	116	2.96e-03
	Insular lobe	Insular gyrus	Ventral agranular insula	Left	165	9.31e-04
				Right	166	1.25e-05
			ventral dysgranular and granular insula	Left	169	1.07e-02
				Right	170	2.63e-05
	Limbic lobe	Cingulate gyrus	Pregenual area 32	Left	179	7.12e-03
			Subgenual area 32	Left	187	9.96e-03
	Subcortical nuclei	Amygdala	Medial amygdala	Left	211	3.86e-04
				Right	212	1.60e-05
			Lateral amygdala	Left	213	7.36e-05
				Right	214	2.19e-06
		Hippocampus	Rostral hippocampus	Left	215	8.25e-04
				Right	216	5.30e-05
			Caudal hippocampus	Left	217	8.36e-03
				Right	218	6.82e-04
		Basal ganglia	Globus pallidus	Left	221	6.38e-05
				Right	222	7.62e-08
			Nucleus accumbens	Left	223	3.59e-04
				Right	224	4.44e-05
			Ventromedial putamen	Left	225	6.29e-05
				Right	226	1.81e-05
			Dorsolateral putamen	Left	229	4.46e-06
				Right	230	1.41e-05
		Thalamus	Pre-motor thalamus	Left	233	1.62e-07
				Right	234	1.02e-06
			Sensory thalamus	Left	235	1.60e-06
				Right	236	1.17e-05
			Posterior parietal thalamus	Left	239	2.72e-04
			Lateral pre-frontal thalamus	Left	245	2.84e-06
				Right	246	3.04e-03
	Cerebellum		Anterior lobe (lobules I-IV)	Left	247	2.11e-07
				Right	248	1.07e-06
			Lobule V	Left	249	5.35e-07
				Right	250	8.08e-08
			Lobule VI	Left	251	2.55e-07
				Right	253	6.27e-06
			Vermis of lobule VI		252	2.62e-06
			Crus I (posterior lobe)	Left	254	7.20e-06
				Right	256	9.90e-05
			Vermis of Crus I		255	8.91e-06
			Crus II (posterior lobe)	Left	257	9.75e-07
				Right	259	2.29e-06
			Vermis of Crus II		258	1.65e-06
			Lobule VIIb	Left	260	1.96e-06
				Right	262	1.95e-06
			Vermis of lobule VIIb		261	5.01e-06
			Lobule VIIIa (posterior lobe)	Left	263	4.82e-06
				Right	265	7.49e-06
			Vermis of lobule VIIIa		264	8.82e-08
			Lobule VIIIb	Left	266	6.54e-07
				Right	268	1.06e-05
			Vermis of lobule VIIIb		267	1.69e-06
			Lobule IX (Uvula)	Left	269	2.32e-05
				Right	271	2.40e-04
			Vermis of lobule IX		270	1.32e-06
			Lobule X (Nodulus)	Left	272	3.71e-03
				Right	274	5.07e-05

The demographic, clinical and laboratory characteristics of patients of both clusters are summarized in Table 1. Patients in cluster 2 had more active SLE with higher SLEDAI-2 K scores, and had a higher frequency of psychosis, and cerebrovascular accidents (CVAs). Cluster 2 patients also had a higher frequency of cutaneous manifestations of SLE such as vasculitis, inflammatory type rash and mucosal ulcers. The haemoglobin levels of cluster 2 patients were lower, and the erythrocyte sedimentation rate (ESR) was higher than in cluster 1 patients. With regards to markers of autoimmunity, cluster 2 patients had a higher frequency of positive anti-dsDNA antibody and anti-Smith antibody with higher anti-Smith antibody titers.

When assessing for correlations between FDG uptake in each region and the composite disease activity score (SLEDAI-2 K), irrespective of cluster membership, 58 regions showed significant correlations (R varying between 0.38 and 0.67 in absolute value) after FDR correction (q = 0.05). Linear regressions between FDG uptake in 2 composite VOIs (comprising respectively all regions where cluster 2 > cluster 1 and all regions where cluster 2 < cluster 1) and disease activity scores across the entire sample also revealed significant associations (see Online Resource 6). In the composite VOI where FDG uptake was higher in cluster 2 versus cluster 1, we found a positive correlation (*r* = 0.55, *p* < 0.001) between the relative FDG uptake and the SLEDAI-2 K score. In the other composite VOI (where relative FDG uptake was higher in cluster 1 compared to 2), we found a negative correlation (*r* = −0.53, *p* < 0.001).

## Discussion

In this study we identified a subgroup of NPSLE patients with a distinct FDG PET-CT uptake pattern and clinical phenotype. Hierarchical cluster analysis identified a subgroup of NPSLE patients (cluster 2) with higher metabolism in basal ganglia, thalami, amygdalae, hippocampi, medial temporal lobes, insular cortices and cerebellum and relative hypometabolism in widespread cortical regions including bilateral frontal, parietal, occipital and lateral temporal regions. Patients with this pattern were found to have more severe SLE, reflected by significantly higher SLEDAI-2 K scores compared to patients in cluster 1.

There is existing NPSLE literature that supports the metabolic pattern observed in cluster 2. Cortical hypometabolism is a well-documented feature of NPSLE on FDG PET-CT [7, 8, 18–28], most commonly involving frontal and parietal cortices but also occipital and temporal regions. In addition, several studies have demonstrated hypermetabolism, most commonly seen in the basal ganglia [7, 18, 21, 23, 29, 30], but also in the thalamus [7, 23], hippocampus and orbitofrontal cortex [23, 29]. Abnormal cerebellar metabolism has only been reported in one previous study without mention of whether glucose metabolism was increased or decreased [26]. Although components of the metabolic pattern of cluster 2 have been previously described in NPSLE literature, this is the first study to demonstrate this particular combination of relative hypo- and hypermetabolism in a distinct cluster. Furthermore, this pattern seems to be common, seen in half of patients included in our sample. Cluster analyses of FDG PET brain scans have been employed for other neurological conditions, such as traumatic brain injury [31], Alzheimer’s disease [32, 33] and primary progressive aphasia [34], but to our knowledge this is the first study utilising this technique for FDG PET-CT in NPSLE.

Cluster 2 was characterized by higher disease activity scores and clinical and laboratory markers of severe disease. This was evidenced by higher SLEDAI-2 K scores (driven by higher rates of psychosis, CVA, vasculitis, rash and mucosal ulcers) and higher rates of anti-dsDNA antibody positivity. Cluster 2 patients also exhibited lower haemoglobin levels, higher ESR levels, and more frequent anti-Smith antibody positivity with higher titers. In their study, Ahn and colleagues demonstrated that anti-Smith antibody levels were associated with disease activity in patients with new-onset SLE [35]. Anti-Smith antibodies have also been associated with higher frequencies of psychosis and vasculitis in SLE [36] and have been correlated with evidence of blood brain barrier breakdown [37]. This suggests that anti-Smith antibodies may facilitate the entry of inflammatory mediators into the central nervous system, which could contribute to neuropsychiatric symptoms [37].

The metabolic pattern and raised antibody profiles in cluster 2 supports a possible overlap with autoimmune encephalitis (AIE). Indeed, a similar metabolic pattern has been described in autoimmune encephalitis (AIE) [38] and multiple cases with co-existing AIE and SLE have been reported, the majority being limbic and striatal encephalitis, suggesting a possible link between the two diseases [39–41]. A recent systematic review on this topic highlights the difficulty in separating these two entities, as symptoms and auto-antibodies between AIE and NPSLE overlap [41]. An auto-antibody of particular interest shared by NPSLE and AIE is the antibody against the N-methyl-D- aspartate receptor (anti-NMDAR antibody) [42]. It is associated with the most common form of AIE, NMDAR-antibody encephalitis [38] and is not routinely tested in NPSLE patients. Interestingly, anti-NR2 antibodies (antibodies against the NR2 subunit of the N-methyl D-aspartate receptor) have been shown to be elevated in the cerebrospinal fluid of NPSLE patients with diffuse manifestations of the disease compared to patients with focal symptoms [43] which would support the non-thrombotic pathophysiological mechanisms thought to be at play in this patient subgroup [39]. Growing evidence supports the hypothesis that anti-dsDNA antibodies, one of the most specific antibodies in SLE, gain access to the CNS and cross-react with neuronal anti-NMDAR antibodies which have been associated with neuropsychiatric symptoms in SLE patients ([44–51]). A study by Kowal et al. demonstrated that these antibodies bind to both DNA and NMDAR receptors and cause neuronal loss in the hippocampus in a murine model [44]. The binding of anti-dsDNA antibodies to NMDAR antigens triggers neuronal excitotoxicity, which manifests as a non-thrombotic, non-vasculitic form of NPSLE [39]. NR2 receptors are found in the hippocampus, the amygdala, anterior hypothalamus, cerebellum and the basal ganglia [52], ostensibly matching the cluster 2 metabolic pattern observed in our study. Anti-dsDNA antibody levels in NPSLE have also been linked to neuropsychiatric symptoms and MRI findings of striatal inflammation [39], with patients in that case series demonstrating an early response to standard therapy for traditional anti-NMDAR AIE leading the authors to postulate that this encephalitic entity of NSPLE is under-recognised [53]. It remains unclear however, whether NPSLE forms part of the AIE spectrum caused by SLE-related autoantibodies or if an alternative pathophysiological mechanism is at play which links these two diseases [41].

Major strengths of this study included the use of data-driven clustering to identify a distinct metabolic pattern in NPSLE and good clinical characterization of participants despite the experiment’s retrospective design. No quantitative measures of PET-CT image quality were performed as part of this retrospective study. Instead, we relied on standard qualitative measures of image quality (including checks for patient motion) as per clinical workflows. While this may be seen as a limitation, it may be argued that there is value in utilizing such data, acquired in a clinical setting in a challenging patient population. Another limitation of this study is that there was no healthy control data, therefore the clinical and imaging phenotype of cluster 1 patients could only be characterized in relation to cluster 2. Although sample size may be criticized, it is arguably defensible given the rarity of NPSLE. It may be that with a larger sample size, additional clusters might have emerged. The lack of a healthy control group and clinical follow up data mean that diagnostic and prognostic implications could not be tested, and these will be subjects of further research.

Prospective studies with more rigorous clinical phenotyping and immunological profiling, including FDG PET at several time points and outcome measures would be valuable in better elucidating subtypes of NSPLE and prognosticating outcomes and may inform future research on targeted interventions.

In conclusion, using a hierarchical clustering analysis we identified a subgroup of NPSLE patients with a distinct FDG PET-CT uptake pattern, higher disease activity and clinical and laboratory markers of severe disease. Metabolic imaging findings support a putative link between NPSLE and AIE with this imaging pattern potentially representing an additional biomarker for disease severity in NPSLE. More research is needed to explore potential diagnostic, prognostic and treatment implications of different NPSLE metabolic patterns.

## Supplementary Information

Below is the link to the electronic supplementary material.

Supplementary file1 (PDF 1.64 MB)

Supplementary file2 (TXT 3.43 KB)

Supplementary file3 (TXT 5.64 KB)

Supplementary file4 (TXT 2.97 KB)

Supplementary file5 (PDF 594 KB)

Supplementary file6 (PDF 231 KB)

Supplementary file7 (DOCX 33.0 KB)

## Data Availability

The datasets generated during and/or analysed during the current study are available from the corresponding author on reasonable request.
