# Supplementary material for: Distinct metabolic patterns of neuropsychiatric systemic lupus erythematosus on hierarchical cluster analysis
Source: Eur J Nucl Med Mol Imaging. 2025 Jun 10;52(13):5085–97. doi: 10.1007/s00259-025-07391-z (PMC12589281; doi:10.1007/s00259-025-07391-z)
Supplement: Supplementary file 1 — Supplementary file1 (PDF 1.64 MB) [file 259_2025_7391_MOESM1_ESM.pdf]

Supplementary Information:

European Journal of Nuclear Medicine and Molecular Imaging

Distinct metabolic patterns of neuropsychiatric systemic lupus erythematosus on hierarchical cluster analysis

Bianca Dagmar Berndorfler[1] (ORCID: 0000-0002-9165-9876)
James Mathew Warwick[1] (ORCID: 0000-0002-2810-0543)
Patrick Dupont[2] (ORCID: 0000-0003-1980-2540)
Riette du Toit[3] (ORCID: 0000-0001-9863-3102)
Amori Engelbrecht[3]
Thabiet Jardine[3] (ORCID: 0000-0003-0305-4891)
Prabash Sadhai[3]
Tholakele Sabela[3]
Vivian Anopuechi-Clarkson[3]
Alex Govert George Doruyter[1,4] (ORCID: 0000-0001-9294-1737)

Affiliations
[1]Division of Nuclear Medicine, Faculty of Medicine and Health Sciences, Stellenbosch University, Cape Town, South Africa.
[3]Department of Neurosciences, KU Leuven Brain Institute, Leuven, Belgium.
[4]Division of Rheumatology, Department of Medicine, Stellenbosch University, Cape Town, South Africa.
[4]NuMeRI Node for Infection Imaging, Central Analytical Facilities, Stellenbosch University, Cape Town, South Africa.

Corresponding author: Bianca D Berndorfler (bibi@sun.ac.za)

Online resource 1: Additional analyses performed

Two additional analyses were performed to assess robustness of our methods:

1. Our original analysis was performed using only the AAL atlas for region segmentation
2. Our original segmentation using the Brainnetome and AAL atlas was used, but a k-means clustering method was employed

The dice coefficients between these methods and our original analysis demonstrates good agreement and therefore proves robustness of our original clustering analyses.

dice coefficient cluster 1 between original and AAL with k=2	0.88
dice coefficient cluster 2 between original and AAL with k=2	0.90
dice coefficient cluster 1 between original and k-means with k=2	0.89
dice coefficient cluster 2 between original and k-means with k=2	0.89

Visualisation of the average FDG images

- Images are normalized so that the average value in voxels belonging to the atlas (=brain) is 100.
- Figure 2 in the paper show slices at

  z = +44mm

  z = +4mm

  z = -33mm

Mean images (voxel-based) of the clusters, original analysis

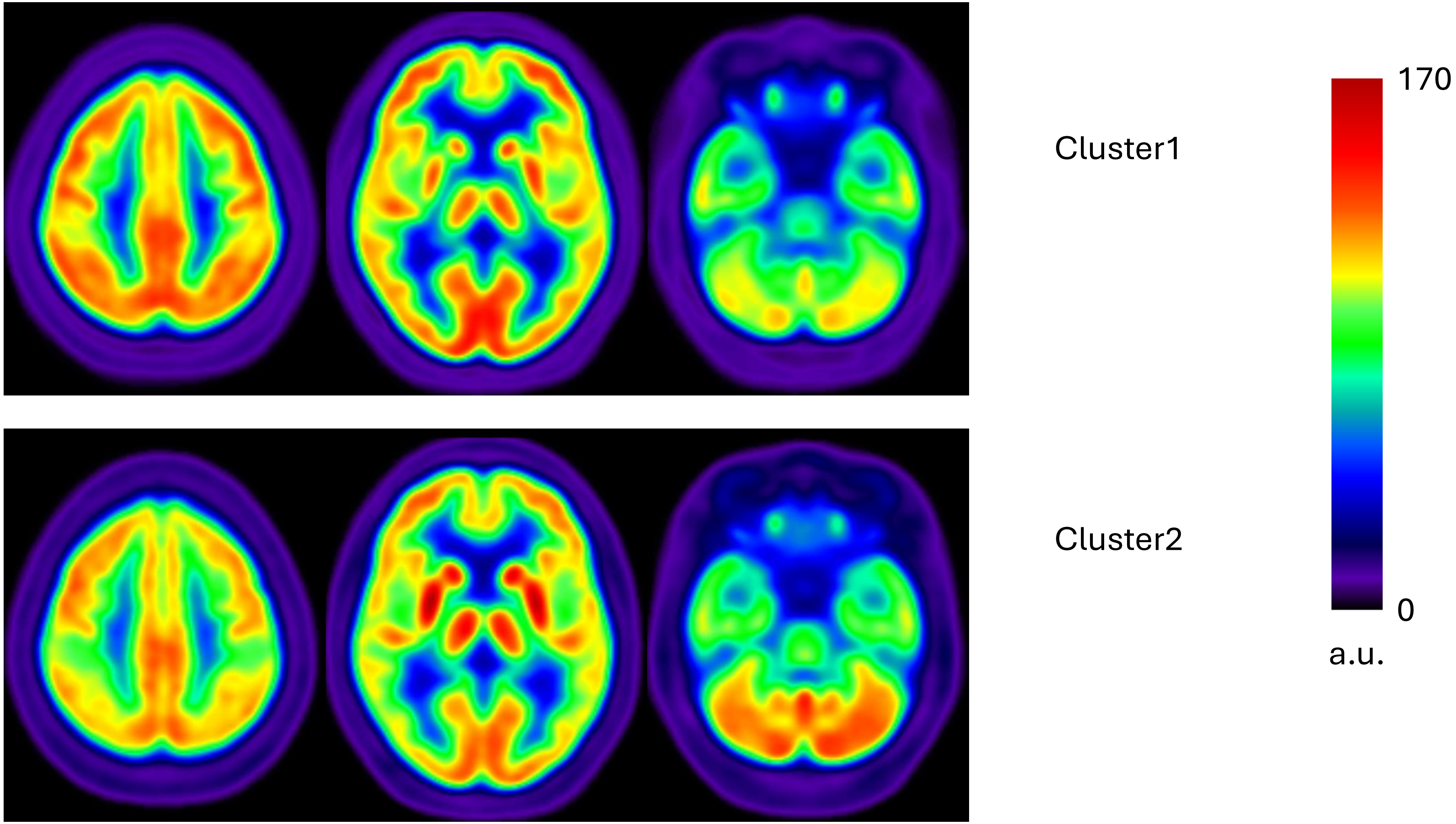

Mean images (voxel-based) of the clusters, using the AAL atlas

(equivalent to figure2 of original analysis)

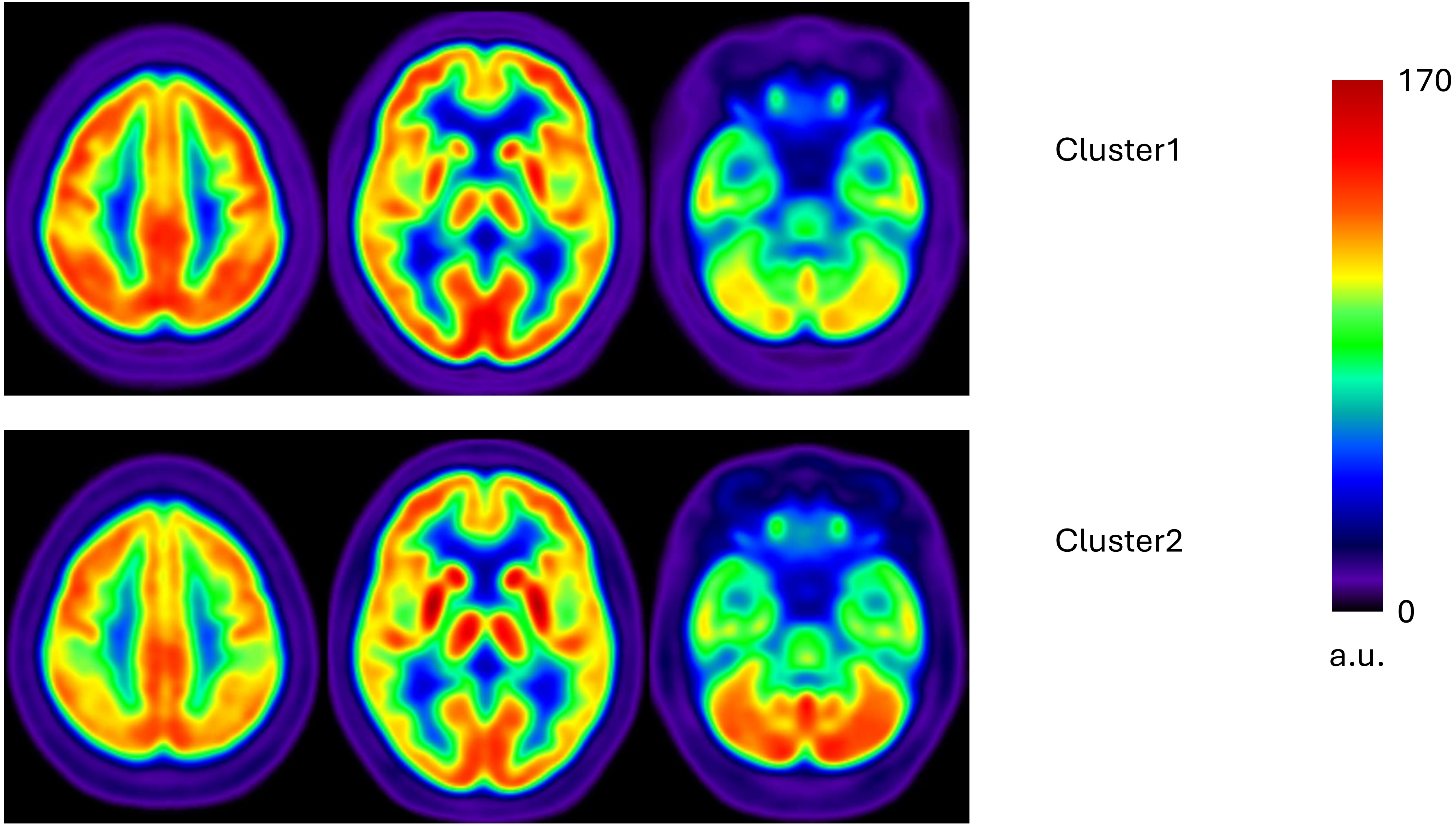

Mean images (voxel-based) of the clusters, using k-means clustering (equivalent to figure2 of original analysis)

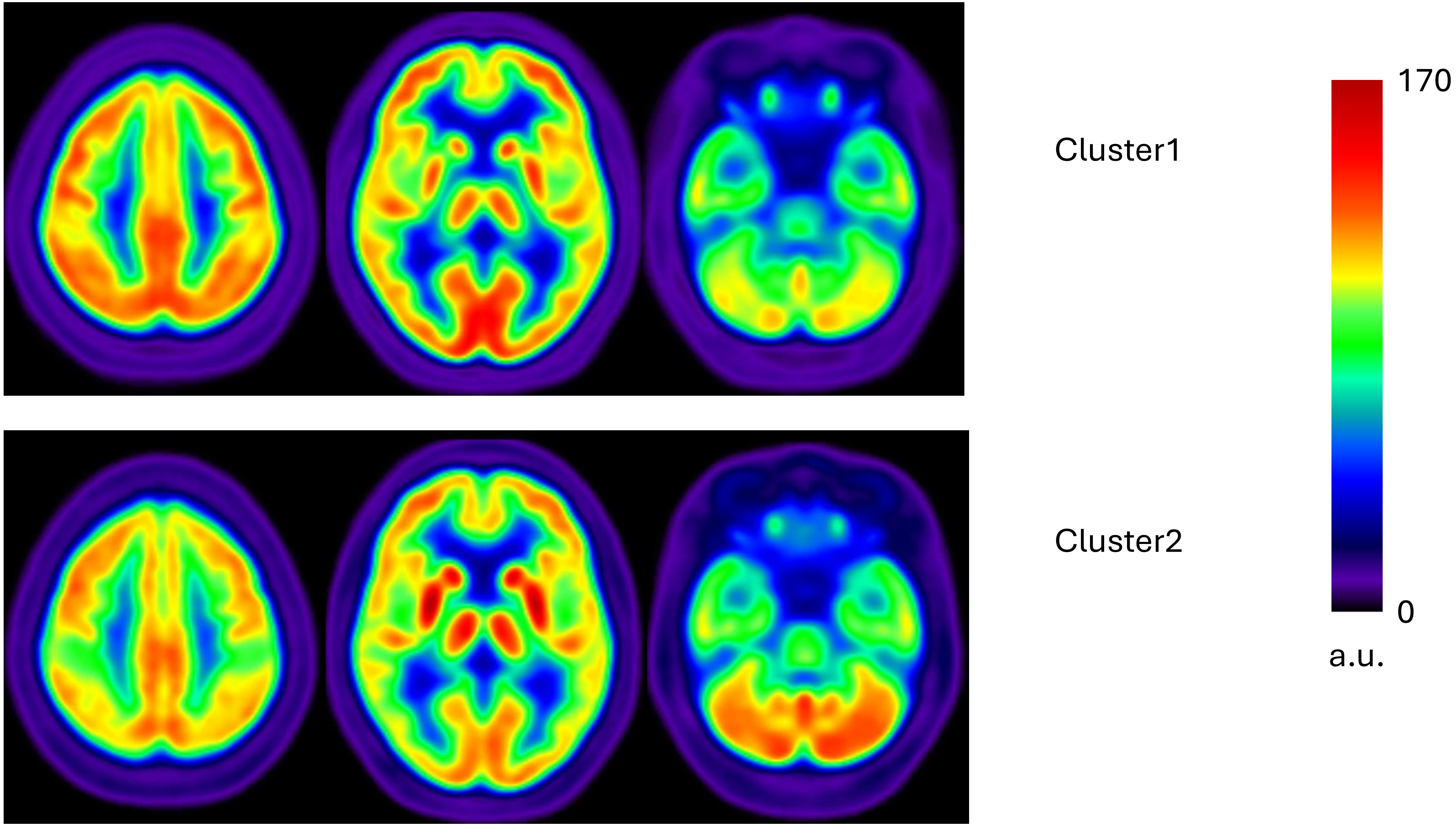

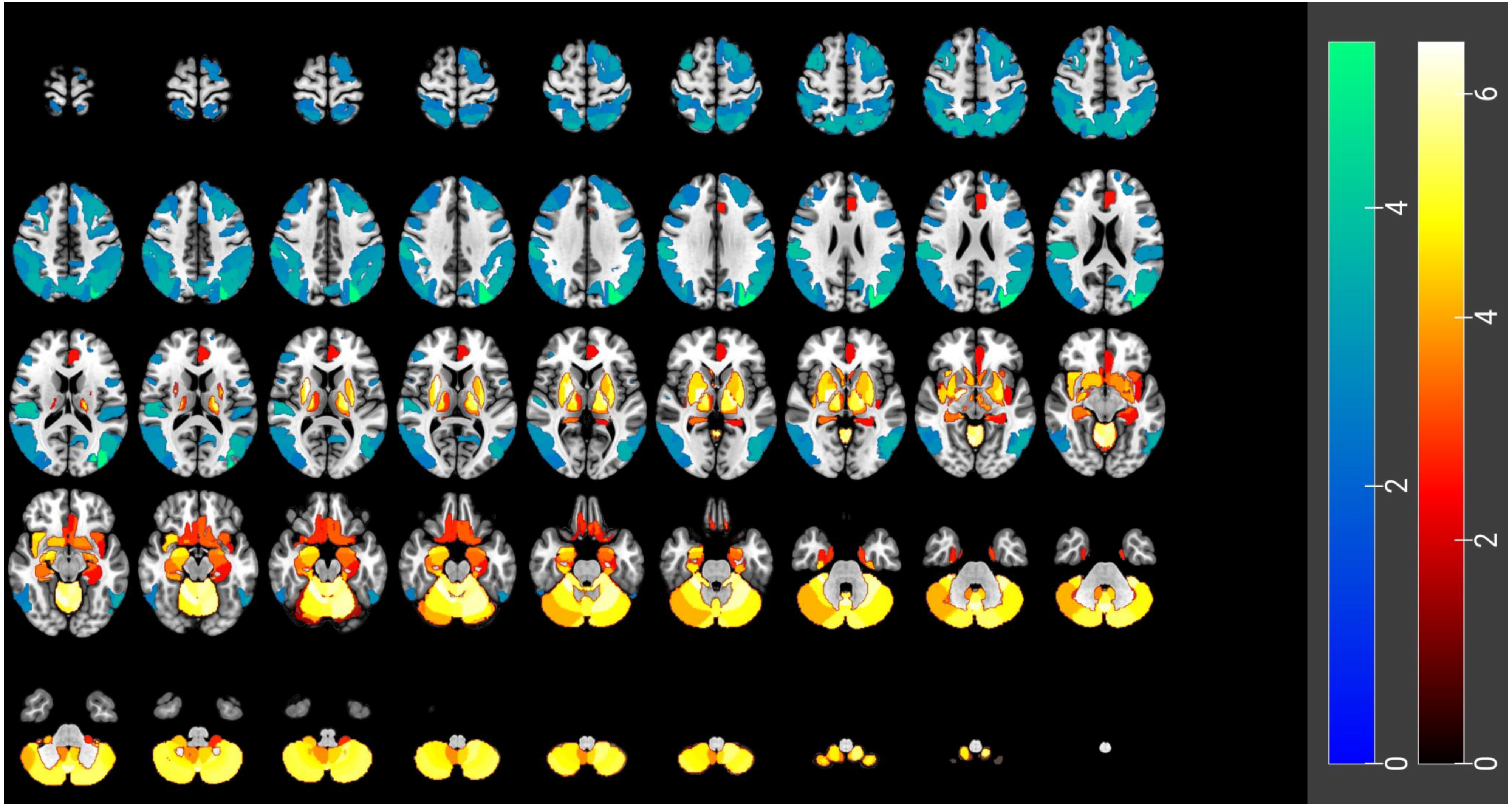

<u>ORIGINAL ANALYSIS statistical results:</u> Statistical region-based comparison showing a significant difference in metabolism (FDR corrected $p < 0.05$), highlighting areas where cluster 2 differed significantly from cluster 1. Increases (red/yellow, “4hot” colour scale) and decreases (blue/turquoise, “5winter” colour scale), overlaid on MRI template (MNI152) in MRIcroGL[53]. The colour scale represents t-values. Abbreviation: FDR = false discovery rate. See ESM2 for detailed output of clustering comparison.

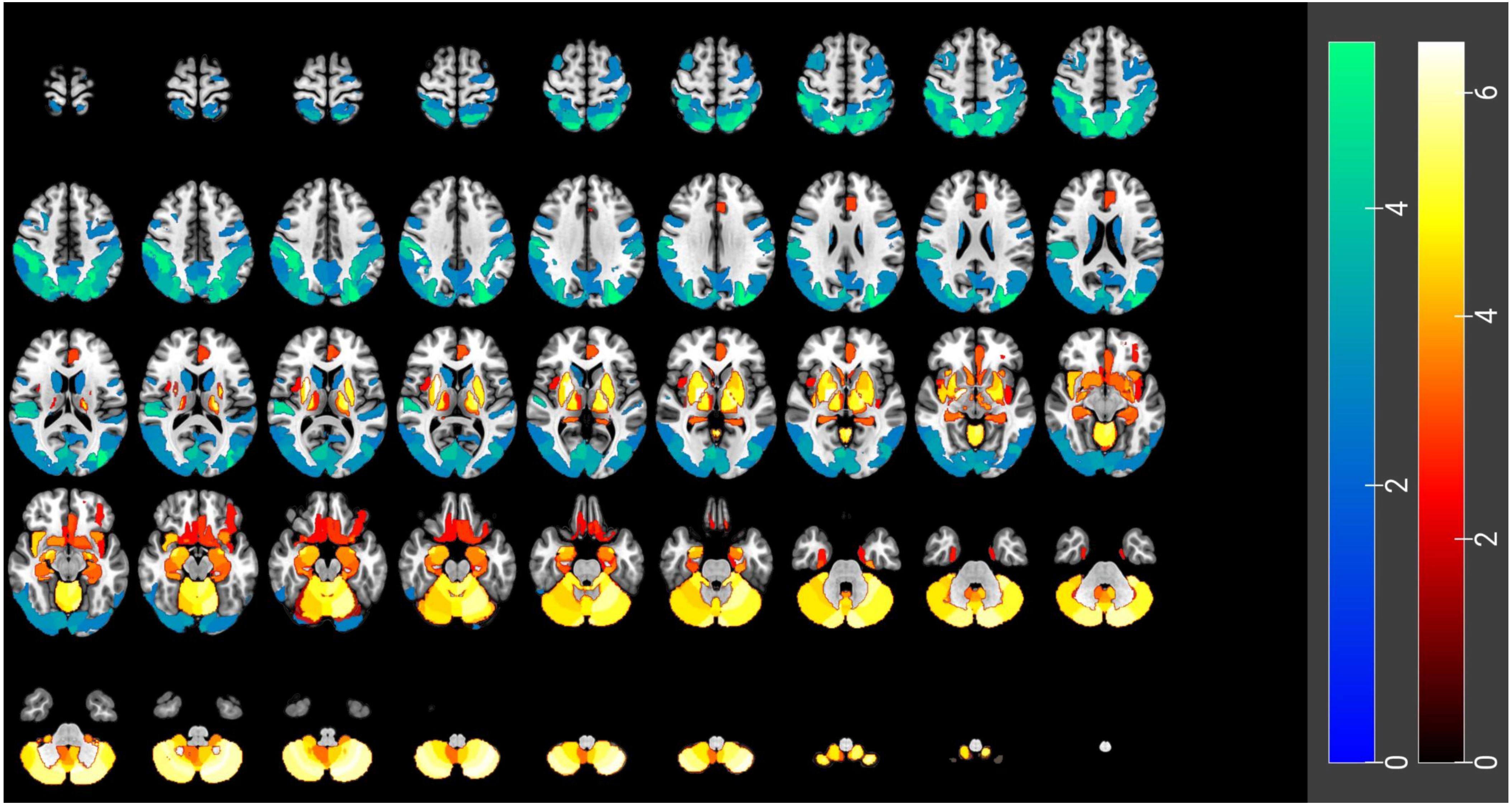

<u>K-MEANS ANALYSIS statistical results</u>: Statistical region-based comparison showing a significant difference in metabolism (FDR corrected $p < 0.05$), highlighting areas where cluster 2 differed significantly from cluster 1. Increases (red/yellow, “4hot” colour scale) and decreases (blue/turquoise, “5winter” colour scale), overlaid on MRI template (MNI152) in MRIcroGL[53]. The colour scale represents t-values. Abbreviation: FDR = false discovery rate. See ESM3 for detailed output of clustering comparison.

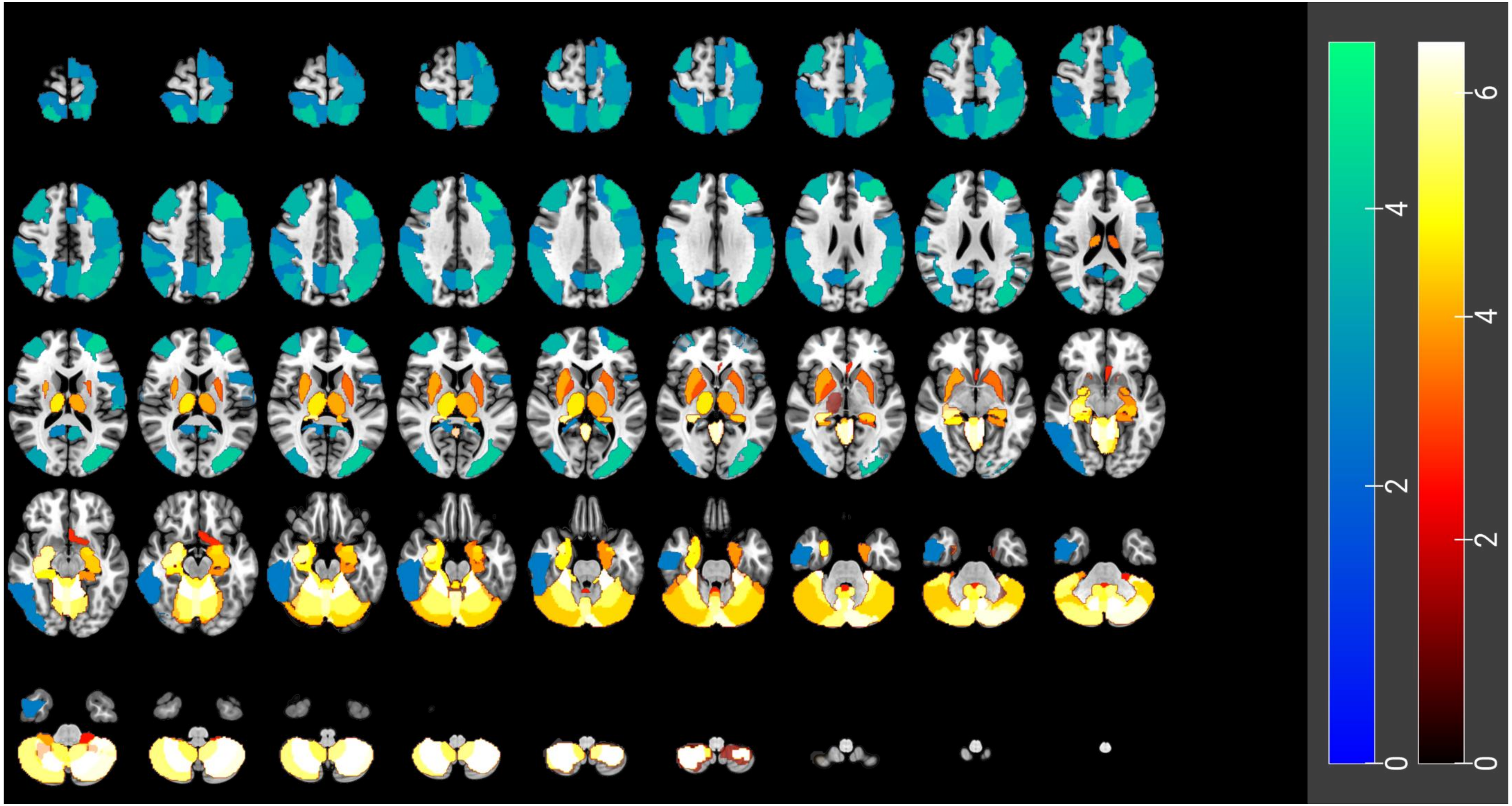

AAL ATLAS ANALYSIS statistical results: Statistical region-based comparison showing a significant difference in metabolism (FDR corrected p < 0.05), highlighting areas where cluster 2 differed significantly from cluster 1. Increases (red/yellow, “4hot” colour scale) and decreases (blue/turquoise, “5winter” colour scale), overlaid on MRI template (MNI152) in MRIcroGL[53]. The colour scale represents t-values. Abbreviation: FDR = false discovery rate. See ESM4 for detailed output of clustering comparison.
