# Supplementary material for: Distinct metabolic patterns of neuropsychiatric systemic lupus erythematosus on hierarchical cluster analysis
Source: Eur J Nucl Med Mol Imaging. 2025 Jun 10;52(13):5085–97. doi: 10.1007/s00259-025-07391-z (PMC12589281; doi:10.1007/s00259-025-07391-z)
Supplement: Supplementary file 5 — Supplementary file5 (PDF 594 KB) [file 259_2025_7391_MOESM5_ESM.pdf]

Supplementary Information:

European Journal of Nuclear Medicine and Molecular Imaging

Distinct metabolic patterns of neuropsychiatric systemic lupus erythematosus on hierarchical cluster analysis

Bianca Dagmar Berndorfler[1] (ORCID: 0000-0002-9165-9876)
James Mathew Warwick[1] (ORCID: 0000-0002-2810-0543)
Patrick Dupont[2] (ORCID: 0000-0003-1980-2540)
Riette du Toit[3] (ORCID: 0000-0001-9863-3102)
Amori Engelbrecht[3]
Thabiet Jardine[3] (ORCID: 0000-0003-0305-4891)
Prabash Sadhai[3]
Tholakele Sabela[3]
Vivian Anopuechi-Clarkson[3]
Alex Govert George Doruyter[1,4] (ORCID: 0000-0001-9294-1737)

Affiliations
[1]Division of Nuclear Medicine, Faculty of Medicine and Health Sciences, Stellenbosch University, Cape Town, South Africa.
[3]Department of Neurosciences, KU Leuven Brain Institute, Leuven, Belgium.
[4]Division of Rheumatology, Department of Medicine, Stellenbosch University, Cape Town, South Africa.
[4]NuMeRI Node for Infection Imaging, Central Analytical Facilities, Stellenbosch University, Cape Town, South Africa.

Corresponding author: Bianca D Berndorfler (bibi@sun.ac.za)

Online resource 5: Optimal number of clusters

- We evaluated k = 2:10 clusters
- Criteria evaluated
  - Elbow method: we calculate the within-cluster sum of squared distances between each point and the centroid of its cluster (WCSS). Then we determine the optimal number of clusters by taking the derivative of the derivative (=acceleration). We obtained k = 2
  - For the gap statistics, the Calinski-Harabasz index, the Silhouette score and the Davies-Bouldin index, we used the evalclusters function in matlab which calculates the optimal k.

Original analysis

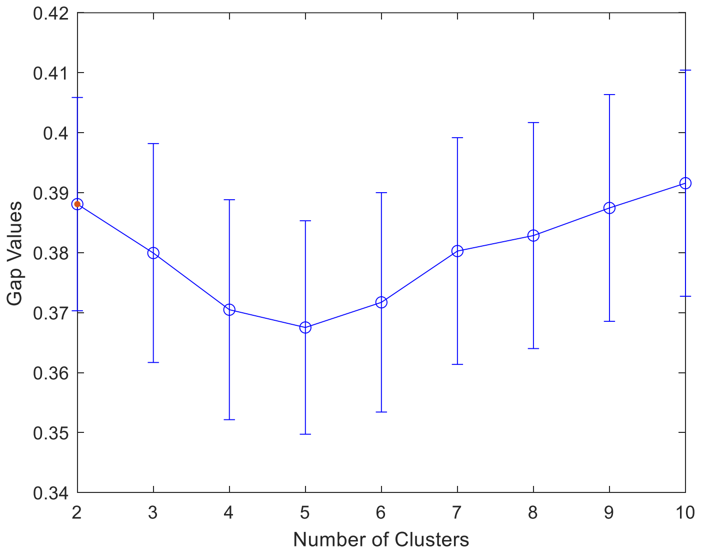

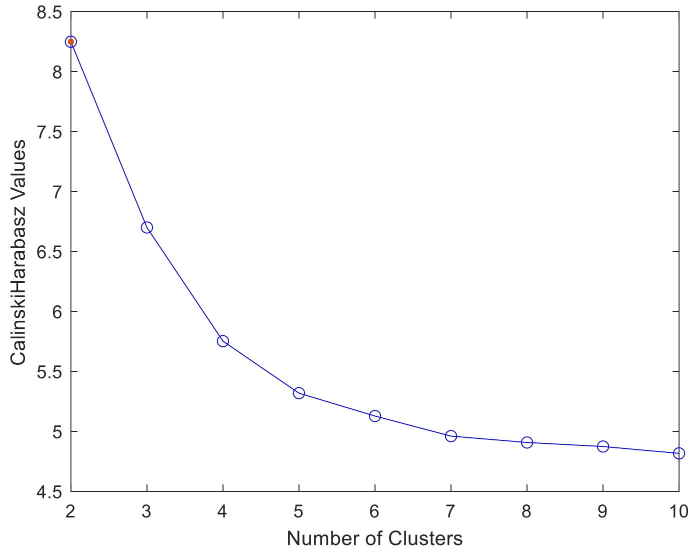

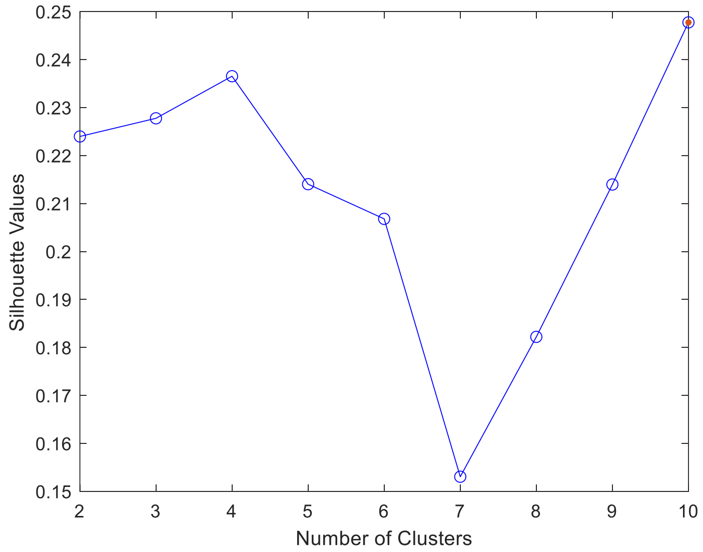

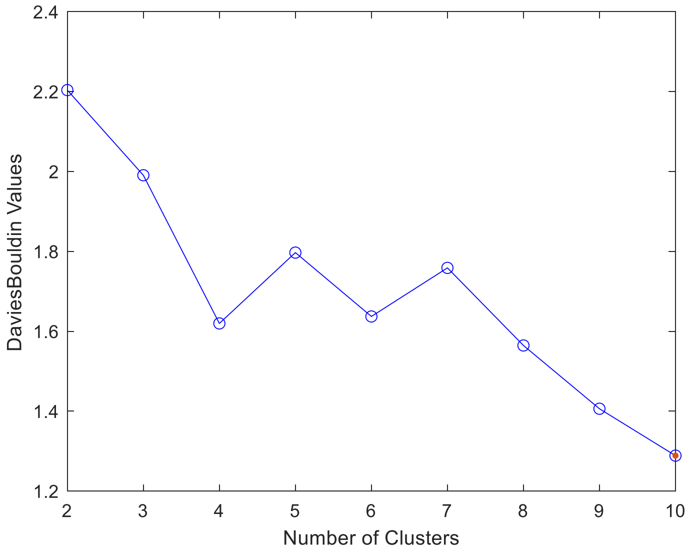

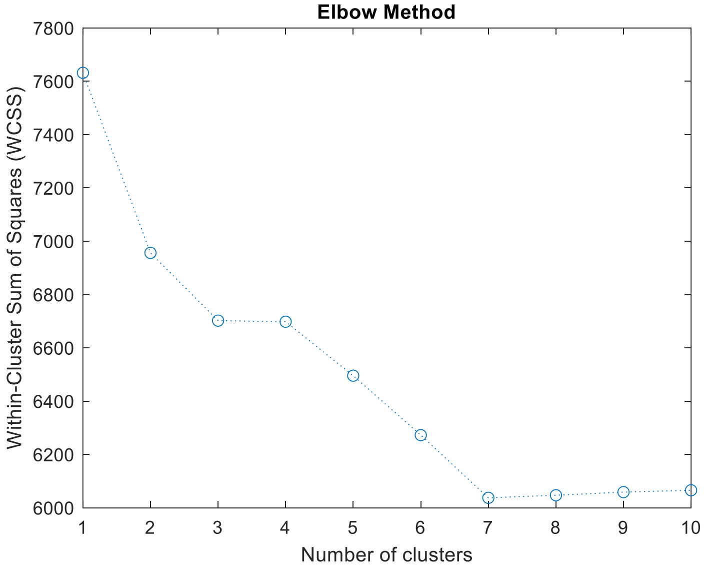

Silhouette Method: optimal number of clusters = 10
Elbow Method: optimal number of clusters = 2
Gap Statistics: optimal number of clusters = 2
Calinski-Harabasz Index: optimal number of clusters = 2
Davies-Bouldin Index: optimal number of clusters = 10

Using the AAL atlas for clustering

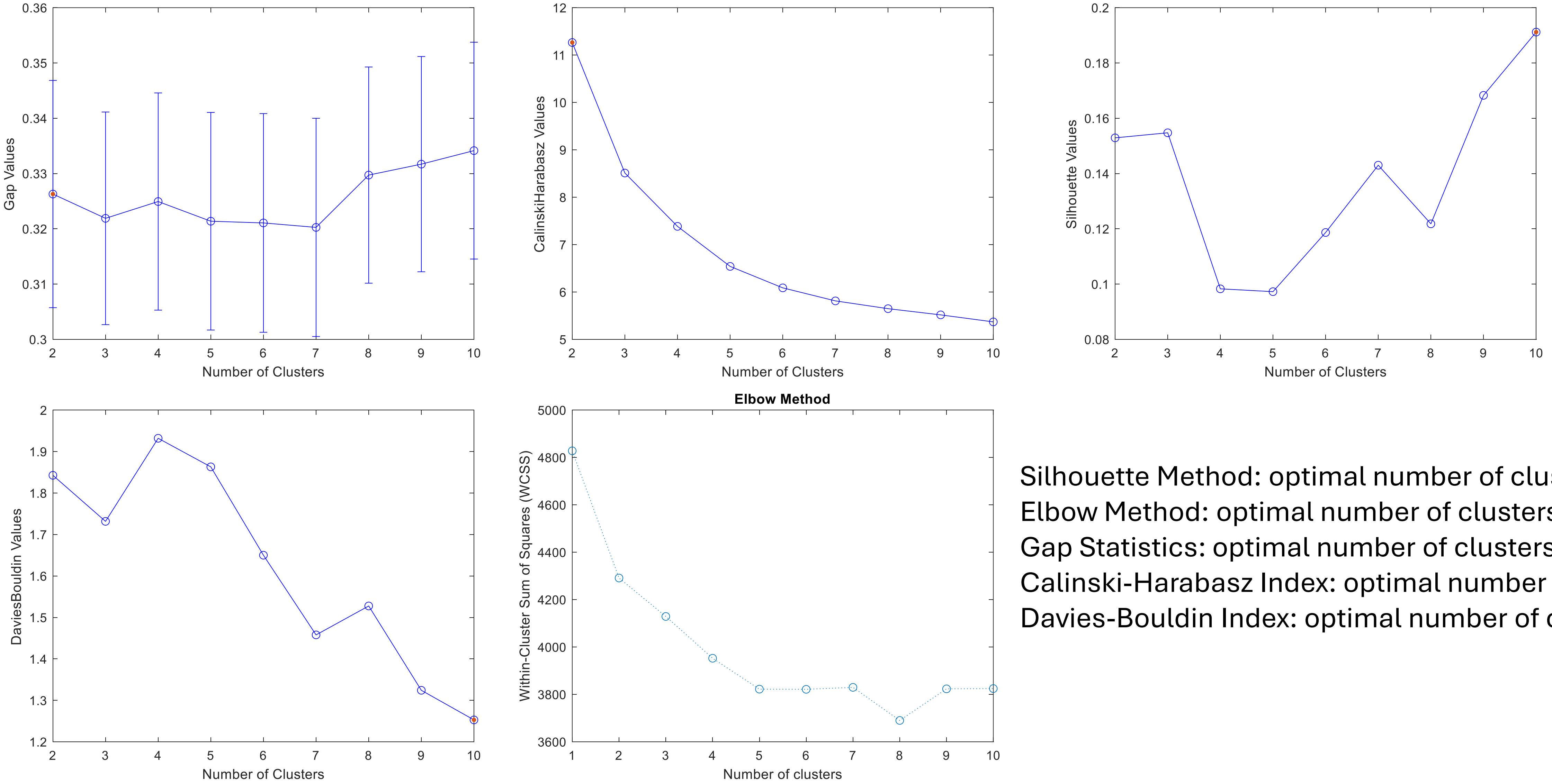

Silhouette Method: optimal number of clusters = 10
Elbow Method: optimal number of clusters = 2
Gap Statistics: optimal number of clusters = 2
Calinski-Harabasz Index: optimal number of clusters = 2
Davies-Bouldin Index: optimal number of clusters = 10

Using k-means clustering

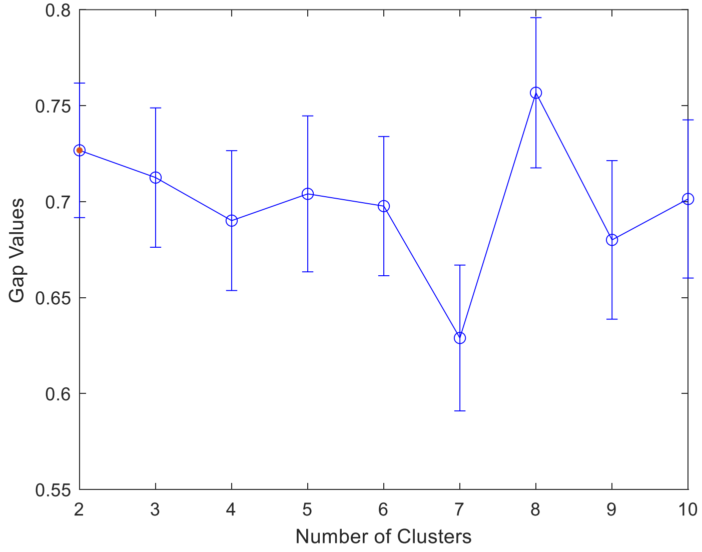

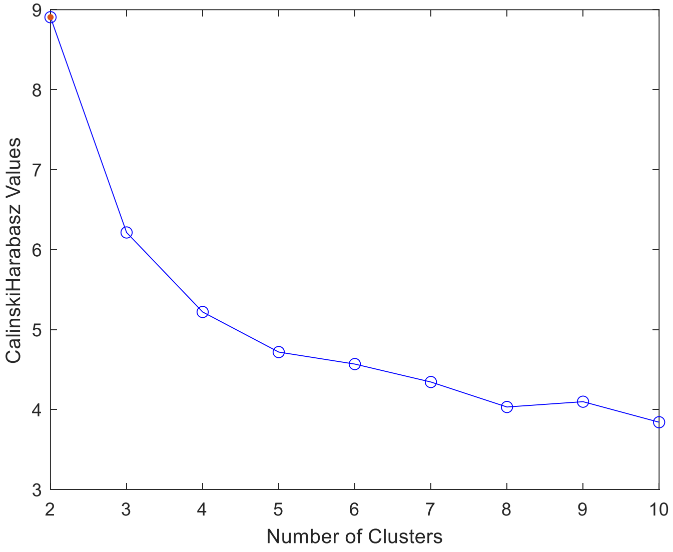

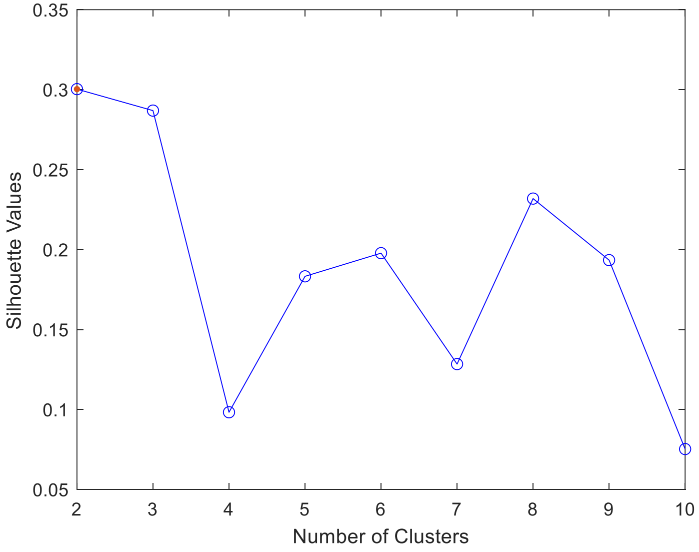

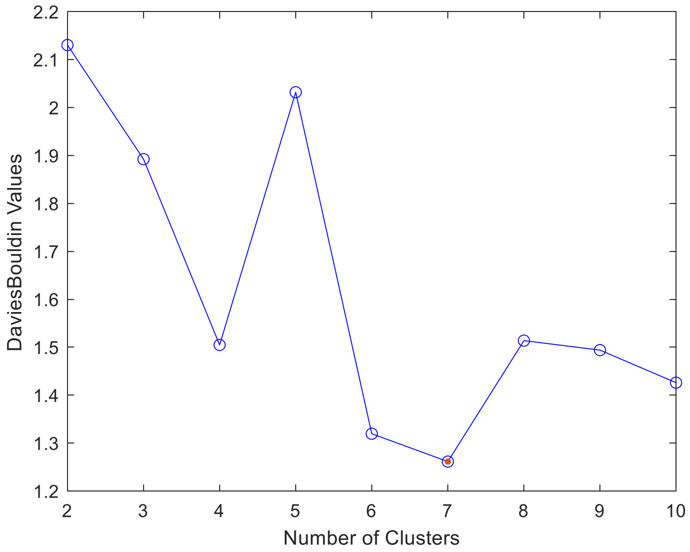

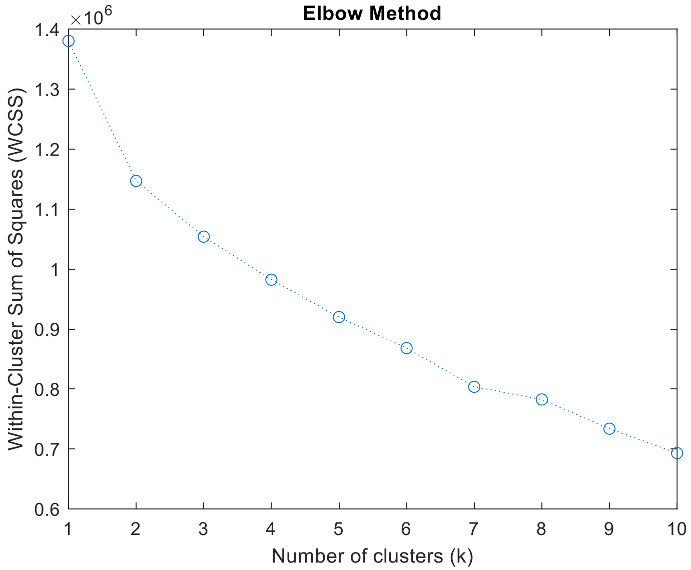

Silhouette Method: optimal number of clusters = 2
Elbow Method: optimal number of clusters = 2
Gap Statistics: optimal number of clusters = 2
Calinski-Harabasz Index: optimal number of clusters = 2
Davies-Bouldin Index: optimal number of clusters = 7
