# Supplementary material for: Distinct metabolic patterns of neuropsychiatric systemic lupus erythematosus on hierarchical cluster analysis
Source: Eur J Nucl Med Mol Imaging. 2025 Jun 10;52(13):5085–97. doi: 10.1007/s00259-025-07391-z (PMC12589281; doi:10.1007/s00259-025-07391-z)

Supplementary Information:

European Journal of Nuclear Medicine and Molecular Imaging

Distinct metabolic patterns of neuropsychiatric systemic lupus erythematosus on hierarchical cluster analysis

Bianca Dagmar Berndorfler[1] (ORCID: 0000-0002-9165-9876)
James Mathew Warwick[1] (ORCID: 0000-0002-2810-0543)
Patrick Dupont[2] (ORCID: 0000-0003-1980-2540)
Riette du Toit[3] (ORCID: 0000-0001-9863-3102)
Amori Engelbrecht[3]
Thabiet Jardine[3] (ORCID: 0000-0003-0305-4891)
Prabash Sadhai[3]
Tholakele Sabela[3]
Vivian Anopuechi-Clarkson[3]
Alex Govert George Doruyter[1,4] (ORCID: 0000-0001-9294-1737)

Affiliations
[1]Division of Nuclear Medicine, Faculty of Medicine and Health Sciences, Stellenbosch University, Cape Town, South Africa.
[3]Department of Neurosciences, KU Leuven Brain Institute, Leuven, Belgium.
[4]Division of Rheumatology, Department of Medicine, Stellenbosch University, Cape Town, South Africa.
[4]NuMeRI Node for Infection Imaging, Central Analytical Facilities, Stellenbosch University, Cape Town, South Africa.

Corresponding author: Bianca D Berndorfler (bibi@sun.ac.za)

Online resource 6: Correlation between FDG uptake and SLEDAI-2K score

Methods

- For each region in the atlas, we performed a correlation between FDG uptake and the composite disease activity score SLEDAI-2K for all subjects irrespective of cluster membership. We had no information on the SLEDAI-2K for one subject.
- Multiple comparisons were corrected using the False Discovery Rate (FDR) procedure using the Benjamini-Hochberg procedure with a $q < 0.05$.
- We also calculate a linear regression between SLEDAI-2K and the FDG uptake in 2 composite regions defined as those regions where FDG uptake in subjects belonging to cluster 1 was more than the FDG uptake in subjects belonging to cluster 2 (and vice versa).

Negative correlations

region ID	Name	r	uncorrected p (surviving FDR correction)
23	MFG_L_7_5__8vl	-0.67	6.20E-07
143	IPL_L_6_5__39rv	-0.59	1.80E-05
15	MFG_L_7_1__9-46d	-0.58	2.80E-05
5	SFG_L_7_3__9l	-0.58	3.40E-05
3	SFG_L_7_2__8dl	-0.55	9.40E-05
31	IFG_L_6_2__IFS	-0.54	1.10E-04
141	IPL_L_6_4__40c	-0.54	1.30E-04
1	SFG_L_7_1__8m	-0.51	3.50E-04
6	SFG_R_7_3__9l	-0.49	7.10E-04
17	MFG_L_7_2__IFJ	-0.48	7.40E-04
11	SFG_L_7_6__9m	-0.48	7.80E-04
25	MFG_L_7_6__6vl	-0.48	8.40E-04
24	MFG_R_7_5__8vl	-0.47	1.10E-03
16	MFG_R_7_1__9-46d	-0.45	1.70E-03
19	MFG_L_7_3__46	-0.44	2.50E-03
139	IPL_L_6_3__40rd	-0.43	2.90E-03
107	FuG_L_3_3__37lv	-0.43	3.10E-03
4	SFG_R_7_2__8dl	-0.43	3.10E-03
55	PrG_L_6_2__6cdl	-0.43	3.50E-03
7	SFG_L_7_4__6dl	-0.41	5.60E-03
32	IFG_R_6_2__IFS	-0.41	5.70E-03
26	MFG_R_7_6__6vl	-0.40	5.90E-03
85	MTG_L_4_3__37dl	-0.40	6.00E-03
145	IPL_L_6_6__40rv	-0.39	7.50E-03
97	ITG_L_7_5__37vl	-0.39	8.60E-03

Positive correlations

region ID	Name	r	uncorrected p (surviving FDR correction)
78	STG_R_6_5__38l	0.38	1.00E-02
233	Tha_L_8_2__mPMtha	0.39	7.90E-03
224	Str_R_6_3__NAC	0.40	5.90E-03
254	Cb_Left_Crus_I	0.40	6.50E-03
70	STG_R_6_1__38m	0.41	5.10E-03
166	INS_R_6_2__vIa	0.41	5.80E-03
255	Cb_Vermis_Crus_I	0.42	4.50E-03
256	Cb_Right_Crus_I	0.43	3.20E-03
268	Cb_Right_VIIIb	0.43	3.30E-03
221	Str_L_6_2__GP	0.43	3.40E-03
263	Cb_Left_VIIIa	0.43	3.50E-03
265	Cb_Right_VIIIa	0.43	3.60E-03
257	Cb_Left_Crus_II	0.44	2.40E-03
248	Cb_Right_I-IV	0.44	2.60E-03
182	CG_R_7_4__23v	0.44	2.80E-03
262	Cb_Right_VIIb	0.45	1.80E-03
241	Tha_L_8_6__Otha	0.45	2.10E-03
259	Cb_Right_Crus_II	0.47	1.00E-03
269	Cb_Left_IX	0.47	1.10E-03
266	Cb_Left_VIIIb	0.47	1.10E-03
222	Str_R_6_2__GP	0.47	1.30E-03
261	Cb_Vermis_VIIb	0.48	8.20E-04
267	Cb_Vermis_VIIIb	0.48	8.30E-04
260	Cb_Left_VIIb	0.48	8.70E-04
247	Cb_Left_I-IV	0.48	9.00E-04
212	Amyg_R_2_1__mAmyg	0.49	6.00E-04
251	Cb_Left_VI	0.50	5.00E-04
264	Cb_Vermis_VIIIa	0.50	5.10E-04
249	Cb_Left_V	0.53	1.90E-04
252	Cb_Vermis_VI	0.53	2.00E-04
253	Cb_Right_VI	0.57	4.20E-05
258	Cb_Vermis_Crus_II	0.58	2.70E-05
250	Cb_Right_V	0.60	1.20E-05

Correlation between SLEDAI and FDG uptake in the composite VOI (all regions where FDG uptake in cluster1 > cluster2)

Estimated Coefficients:

	Estimate	SE	tStat	pValue
(Intercept)	146.7	29.865	4.9123	1.3485e-05
x1	-1.1766	0.28358	-4.1489	0.00015459

Number of observations: 45, Error degrees of freedom: 43

- Root Mean Squared Error: 8.61
- R-squared: 0.286, Adjusted R-Squared: 0.269
- F-statistic vs. constant model: 17.2, p-value = 0.000155

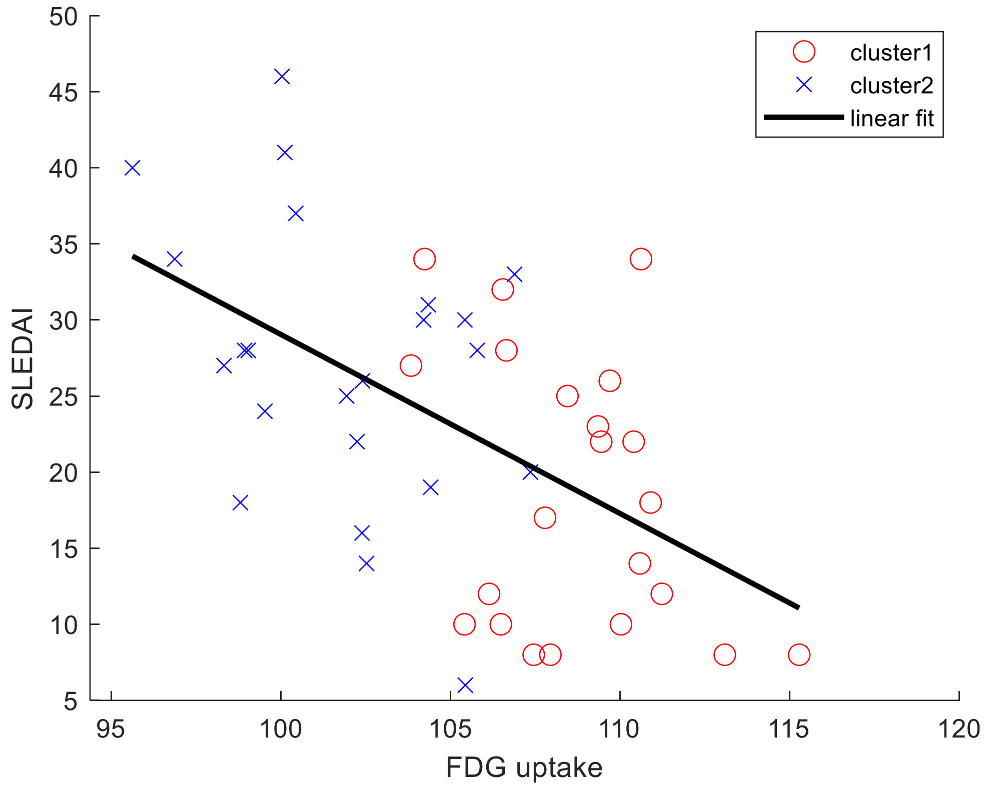

Correlation between SLEDAI and FDG uptake in the composite VOI (all regions where FDG uptake in cluster2 > cluster1)

Estimated Coefficients:

Estimated Coefficients:

	Estimate	SE	tStat	pValue
(Intercept)	-42.426	15.346	-2.7647	0.0083572
x1	0.67248	0.1574	4.2725	0.00010503

Number of observations: 45, Error degrees of freedom: 43

Root Mean Squared Error: 8.54

R-squared: 0.298, Adjusted R-Squared: 0.282

F-statistic vs. constant model: 18.3, p-value = 0.000105

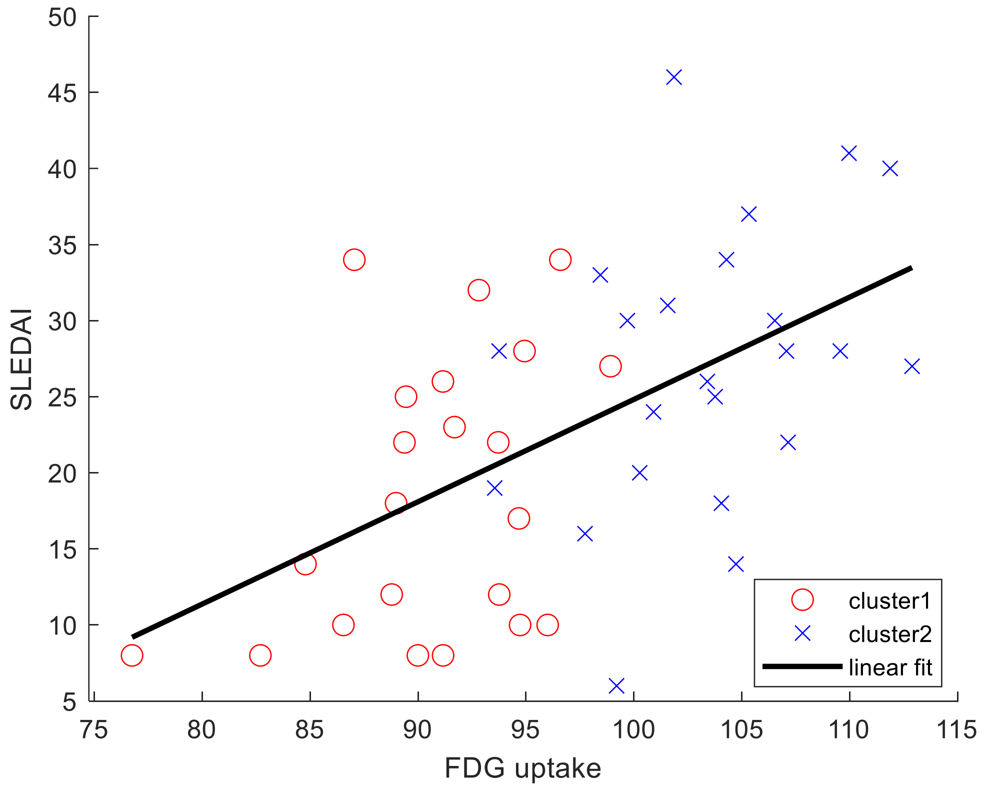

Supplement: Supplementary file 6 — Supplementary file6 (PDF 231 KB) [file 259_2025_7391_MOESM6_ESM.pdf]
