# Supplementary material for: Distinct metabolic patterns of neuropsychiatric systemic lupus erythematosus on hierarchical cluster analysis
Source: Eur J Nucl Med Mol Imaging. 2025 Jun 10;52(13):5085–97. doi: 10.1007/s00259-025-07391-z (PMC12589281; doi:10.1007/s00259-025-07391-z)
Supplement: Supplementary file 7 — Supplementary file7 (DOCX 33.0 KB) [file 259_2025_7391_MOESM7_ESM.docx]

Supplementary information

| Contrast | Region label ID | Descriptor |
| --- | --- | --- |
| Cluster 2 < cluster 1 | 1 | SFG_L_7_1__8m |
|  | 3 | SFG_L_7_2__8dl |
|  | 5 | SFG_L_7_3__9l |
|  | 7 | SFG_L_7_4__6dl |
|  | 15 | MFG_L_7_1__9-46d |
|  | 16 | MFG_R_7_1__9-46d |
|  | 23 | MFG_L_7_5__8vl |
|  | 24 | MFG_R_7_5__8vl |
|  | 25 | MFG_L_7_6__6vl |
|  | 26 | MFG_R_7_6__6vl |
|  | 29 | IFG_L_6_1__44d |
|  | 32 | IFG_R_6_2__IFS |
|  | 55 | PrG_L_6_2__6cdl |
|  | 63 | PrG_L_6_6__6cvl |
|  | 64 | PrG_R_6_6__6cvl |
|  | 72 | STG_R_6_2__41-42 |
|  | 85 | MTG_L_4_3__37dl |
|  | 86 | MTG_R_4_3__37dl |
|  | 91 | ITG_L_7_2__37elv |
|  | 92 | ITG_R_7_2__37elv |
|  | 97 | ITG_L_7_5__37vl |
|  | 98 | ITG_R_7_5__37vl |
|  | 125 | SPL_L_5_1__7r |
|  | 126 | SPL_R_5_1__7r |
|  | 127 | SPL_L_5_2__7c |
|  | 128 | SPL_R_5_2__7c |
|  | 129 | SPL_L_5_3__5l |
|  | 130 | SPL_R_5_3__5l |
|  | 131 | SPL_L_5_4__7pc |
|  | 132 | SPL_R_5_4__7pc |
|  | 133 | SPL_L_5_5__7ip |
|  | 135 | IPL_L_6_1__39c |
|  | 136 | IPL_R_6_1__39c |
|  | 137 | IPL_L_6_2__39rd |
|  | 138 | IPL_R_6_2__39rd |
|  | 139 | IPL_L_6_3__40rd |
|  | 140 | IPL_R_6_3__40rd |
|  | 141 | IPL_L_6_4__40c |
|  | 142 | IPL_R_6_4__40c |
|  | 143 | IPL_L_6_5__39rv |
|  | 144 | IPL_R_6_5__39rv |
|  | 145 | IPL_L_6_6__40rv |
|  | 146 | IPL_R_6_6__40rv |
|  | 147 | PCun_L_4_1__7m |
|  | 148 | PCun_R_4_1__7m |
|  | 149 | PCun_L_4_2__5m |
|  | 151 | PCun_L_4_3__dmPOS |
|  | 159 | PoG_L_4_3__2 |
|  | 160 | PoG_R_4_3__2 |
|  | 200 | OcG_R_4_1__mOccG |
|  | 201 | OcG_L_4_2__V5-MT |
|  | 202 | OcG_R_4_2__V5-MT |
|  | 209 | sOcG_L_2_2__lsOccG |
|  | 210 | sOcG_R_2_2__lsOccG |
| Cluster 2 > cluster 1 | 49 | OrG_L_6_5__13 |
|  | 50 | OrG_R_6_5__13 |
|  | 110 | PhG_R_6_1__35-36r |
|  | 111 | PhG_L_6_2__35-36c |
|  | 112 | PhG_R_6_2__35-36c |
|  | 113 | PhG_L_6_3__TL |
|  | 114 | PhG_R_6_3__TL |
|  | 115 | PhG_L_6_4__28-34 |
|  | 116 | PhG_R_6_4__28-34 |
|  | 165 | INS_L_6_2__vIa |
|  | 166 | INS_R_6_2__vIa |
|  | 169 | INS_L_6_4__vId-vIg |
|  | 170 | INS_R_6_4__vId-vIg |
|  | 179 | CG_L_7_3__32p |
|  | 187 | CG_L_7_7__32sg |
|  | 211 | Amyg_L_2_1__mAmyg |
|  | 212 | Amyg_R_2_1__mAmyg |
|  | 213 | Amyg_L_2_2__lAmyg |
|  | 214 | Amyg_R_2_2__lAmyg |
|  | 215 | Hipp_L_2_1__rHipp |
|  | 216 | Hipp_R_2_1__rHipp |
|  | 217 | Hipp_L_2_2__cHipp |
|  | 218 | Hipp_R_2_2__cHipp |
|  | 221 | Str_L_6_2__GP |
|  | 222 | Str_R_6_2__GP |
|  | 223 | Str_L_6_3__NAC |
|  | 224 | Str_R_6_3__NAC |
|  | 225 | Str_L_6_4__vmPu |
|  | 226 | Str_R_6_4__vmPu |
|  | 229 | Str_L_6_6__dlPu |
|  | 230 | Str_R_6_6__dlPu |
|  | 233 | Tha_L_8_2__mPMtha |
|  | 234 | Tha_R_8_2__mPMtha |
|  | 235 | Tha_L_8_3__Stha |
|  | 236 | Tha_R_8_3__Stha |
|  | 239 | Tha_L_8_5__PPtha |
|  | 245 | Tha_L_8_8__lPFtha |
|  | 246 | Tha_R_8_8__lPFtha |
|  | 247 | Cb_Left_I-IV |
|  | 248 | Cb_Right_I-IV |
|  | 249 | Cb_Left_V |
|  | 250 | Cb_Right_V |
|  | 251 | Cb_Left_VI |
|  | 253 | Cb_Right_VI |
|  | 252 | Cb_Vermis_VI |
|  | 254 | Cb_Left_Crus_I |
|  | 256 | Cb_Right_Crus_I |
|  | 255 | Cb_Vermis_Crus_I |
|  | 257 | Cb_Left_Crus_II |
|  | 259 | Cb_Right_Crus_II |
|  | 258 | Cb_Vermis_Crus_II |
|  | 260 | Cb_Left_VIIb |
|  | 262 | Cb_Right_VIIb |
|  | 261 | Cb_Vermis_VIIb |
|  | 263 | Cb_Left_VIIIa |
|  | 265 | Cb_Right_VIIIa |
|  | 264 | Cb_Vermis_VIIIa |
|  | 266 | Cb_Left_VIIIb |
|  | 268 | Cb_Right_VIIIb |
|  | 267 | Cb_Vermis_VIIIb |
|  | 269 | Cb_Left_IX |
|  | 271 | Cb_Right_IX |
|  | 270 | Cb_Vermis_IX |
|  | 272 | Cb_Left_X |
|  | 274 | Cb_Right_X |

Regions of significant difference in metabolism between cluster 1 and 2 (FDR corrected p < 0.05). Region numbers of the cerebrum refer to the Brainnetome atlas [14] and for the cerebellum the Automated Anatomical Labeling (AAL) [15]
